# Global burden and trends of viral hepatitis among women of childbearing age from 1990 to 2021

**DOI:** 10.3389/fmicb.2025.1553129

**Published:** 2025-02-21

**Authors:** Shaojie Yang, Lin Zhong, Lu Huang, Shengyuan Lin, Yubin Li

**Affiliations:** ^1^Department of Laboratory Medicine, Jiangmen Maternity and Child Health Care Hospital, Jiangmen, Guangdong, China; ^2^Department of Disease Control and Prevention, Jiangmen Municipal Health Bureau, Jiangmen, China

**Keywords:** Global Burden of Disease, viral hepatitis, women of childbearing age, age-standardized rate, estimated annual percentage change

## Abstract

**Background:**

The burden and trends of viral hepatitis in women of childbearing age (WCBA) are rarely quantified. This study aimed to assess the global, regional, and national incidence and prevalence rates among WCBA from 1990 to 2021.

**Methods:**

From 1990 to 2021, we retrieved data from the Global Burden of Diseases, Injuries, and Risk Factors Study (GBD) 2021 on the incidence and prevalence of hepatitis A, B, C, and E for WCBA. Estimated annual percent change in the age-standardized incidence and prevalence rates were calculated to quantify the temporal trend.

**Results:**

In 2021, it was estimated that there were 42,266,708 new cases and 109,107,759 prevalent cases of viral hepatitis among WCBA globally. AHA had the highest incidence rate, while CHB had the highest prevalence rate globally. Notably, AHA and AHE were emerging in low-endemic regions. Generally, the burden of viral hepatitis decreased with higher SDI levels, except for AHA. Between 1990 and 2021, the global age-standardized incidence rate for viral hepatitis among WCBA decreased annually by −1.11% for acute hepatitis A (AHA), −1.24% for AHB, and −0.18% for AHC, −0.34% for AHE, with more significant reductions observed for chronic hepatitis B (CHB) and CHC at −1.33% and −0.29%, respectively. Furthermore, the burden of viral hepatitis continued to rise in lower-SDI regions, and the proportion of younger individuals affected increased as SDI decreased.

**Conclusions:**

Although the incidence and prevalence rates of viral hepatitis have decreased in recent decades, notable regional and demographic disparities remain. These concerning trends are especially pronounced in low-SDI regions, making it essential to tackle the disparities in healthcare resource allocation for WCBA across areas with varying SDI levels.

## 1 Introduction

The World Health Organization's 2024 Global Hepatitis Report highlights the growing threat of viral hepatitis, which caused 1.3 million deaths in 2022 and continues to be a major global health challenge, with 6,000 new infections daily (World Health Organization, 2024). Despite efforts, achieving disease elimination by 2030 remains a distant goal (Cox et al., 2020). Among women of childbearing age (WCBA), viral hepatitis is particularly concerning due to its potential to harm both mother and child, increasing the risk of adverse pregnancy outcomes and mother-to-child transmission (MTCT), which underscores the urgency of addressing this burden (Terrault et al., 2021; 2023).

Hepatitis A infection typically occurs during childhood, often without symptoms, leading to lifelong immunity (Schmutz et al., 2019). However, since the late 1990s, improved living conditions and widespread childhood vaccination have reduced early exposure to the virus, shifting susceptibility to adolescents and adults (Lemon et al., 2018). This shift has made more women of reproductive age vulnerable to hepatitis A, increasing the risk of infection during pregnancy. In South Korea, for instance, a decline in hepatitis A virus (HAV) prevalence among children led to a significant rise in cases among women, with infections jumping from 151 in 2002 to 4,779 in 2009, especially in those aged 20–39 years (Cho et al., 2013). In 2015, the WHO estimated that 257 million people globally were living with chronic hepatitis B, including 65 million women of reproductive age (Tan et al., 2021). In China, which bears the largest burden of hepatitis B virus (HBV) infection, the prevalence among women of reproductive age and pregnant women ranged from 3.87% to 9.98% (Liu et al., 2017). Each year, approximately 4.5 million women with chronic HBV infection give birth, with the majority residing in Africa and the Western Pacific (Terrault et al., 2021). Vertical transmission, especially in regions with high or intermediate HBV prevalence, is responsible for nearly 90% of global chronic HBV cases (MacLachlan and Cowie, 2015). Infants infected in their 1^st^ year of life have an 80%–90% chance of developing chronic infection, compared to just 5% in adults infected later in life (Sheena et al., 2022).

A previous modeling study estimated that nearly 15 million women aged 15–49 were living with hepatitis C virus (HCV) in 2019, representing about 20% of the global HCV-infected population (Dugan et al., 2021). The estimated seroprevalence of HCV in pregnant women (1.72%–3.57%) is about 1.5 times higher than in the general population (1.4%−2.3%) (Abbasi et al., 2023). HCV infection in pregnant women has surged over the past decade, particularly in the United States, where rising opioid use—especially injection drug use—among women of reproductive age is a significant factor (Dugan et al., 2021; Abbasi et al., 2023). Between 2011 and 2016, the prevalence of anti-HCV antibody positivity rose by 36% among WCBA and by 13% among children under 5 in the U.S. (Schillie et al., 2018). Hepatitis D virus (HDV) can only infect individuals already infected with HBV, and over 15 million people globally are co-infected (Hughes et al., 2011). While the exact prevalence of HDV in WCBA is unclear due to limited routine testing in many regions, studies have found that HDV infection affects 14.7% of pregnant women in Mauritania and 20.3% in Pakistan (Terrault et al., 2021). Similar to HAV, hepatitis E virus (HEV) is mainly transmitted through the fecal-oral route in developing countries, often via contaminated drinking water (Aggarwal, 2013). In contrast, in developed countries, HEV transmission commonly occurs through zoonotic sources and is associated with the consumption of poorly cooked, contaminated food (The Lancet Gastroenterology and Hepatology, 2016). HEV seroprevalence among women aged 15 to 45 years is estimated between 5% and 22% (Rein et al., 2012). Infection rates peak in individuals aged 15 to 19 years and decrease with increasing age. Pregnant women face a heightened risk, with case-fatality ratios during the third trimester estimated to be as high as 10%−25%, and a significant incidence of stillbirths (Ciglenecki, 2017).

Preventing MTCT of hepatitis virus is vital for reducing the global burden of viral hepatitis (Terrault et al., 2021). Effective prevention strategies must account for local healthcare and socioeconomic challenges, requiring region-specific approaches that align with regional epidemiology and are backed by national elimination plans and adequate resources (Cox et al., 2020; Dionne-Odom et al., 2022). Despite the importance of this issue, comprehensive estimates of viral hepatitis burden and trends among WCBA are still limited. Our study sought to assess trends in viral hepatitis incidence and prevalence among WCBA from 1990 to 2021 at global, regional, and national levels. This study is the first to provide a comprehensive, national-level analysis of viral hepatitis incidence and prevalence trends among women of childbearing age (WCBA) from 1990 to 2021. Furthermore, our study delves into the impact of the Socio-Demographic Index (SDI) on these trends, offering a more detailed analysis of regional and demographic disparities in the burden of viral hepatitis.

## 2 Methods

### 2.1 Overview

The GBD 2021 is an international collaborative research initiative that offers comprehensive epidemiological data on 371 diseases and injuries, along with 88 attributable risk factors, across 204 countries and territories, as well as 811 subnational locations, broken down by age, sex, and location over time (Ferrari et al., 2024).

### 2.2 Data source

Data on annual viral hepatitis incidence and prevalence from 1990 to 2021 among women of childbearing age (15–49 years) in 204 countries and territories were retrieved using the Global Health Data Exchange (GHDx) query tool (http://ghdx.healthdata.org/gbd-results-tool) (Ferrari et al., 2024). These countries and territories were categorized into 21 GBD regions based on epidemiological similarities and geographic proximity (Supplementary Table 1). Additionally, using the Socio-demographic Index (SDI), these regions and countries were divided into five distinct quintiles: low-, low-middle-, middle-, high-middle-, and high-SDI regions. The study population was defined as women aged 15–49 years, divided into seven GBD age groups at 5-year intervals: 15–19, 20–24, 25–29, 30–34, 35–39, 40–44, and 45–49 years. This study included cases of acute hepatitis A, B, C, and E, as well as chronic hepatitis B and C. In the GBD, anti-hepatitis virus seroprevalence data from population-based studies and surveys served as the primary source for estimating infection incidence (Xiao et al., 2024). The burden of viral hepatitis was estimated using Bayesian meta-regression models (DisMod-MR 2.1), taking into account location, age, sex, year, and pathogen. Uncertainty intervals for each indicator were calculated from the 25th to 95th values of the posterior distribution based on 1,000 iterations. In the GBD 2021, missing data were primarily addressed using Bayesian meta-regression models (DisMod-MR 2.1), which estimate disease burden across multiple dimensions, including location, age, sex, year, and pathogen. These models combine existing data with statistical techniques to fill gaps, drawing from studies with similar epidemiological profiles to generate more reliable estimates. To quantify the uncertainty around these estimates, uncertainty intervals were calculated from the posterior distribution based on 1,000 iterations. For countries with incomplete data, imputation and extrapolation methods were employed to handle the missing values, utilizing patterns from available data in similar regions or countries. The uncertainty associated with these estimates was again quantified through posterior distribution intervals. Detailed information on the estimated incidence and prevalence of hepatitis, including data input, processing, and modeling methods, has been described in previous studies (Ferrari et al., 2024).

### 2.3 Case definition

In GBD 2021, acute hepatitis A (AHA) was defined as an infection with the hepatitis A virus (HAV) that leads to anti-HAV IgG seroconversion, irrespective of symptoms. Acute hepatitis B (AHB) was characterized as the initial phase of infection with the hepatitis B virus, regardless of symptoms. Similarly, acute hepatitis C (AHC) was defined as the initial phase of infection with the hepatitis C virus (HCV), resulting in anti-HCV IgG seroconversion, regardless of symptoms. Acute hepatitis E (AHE) was defined as an infection with the hepatitis E virus (HEV) that results in anti-HEV IgG seroconversion, irrespective of symptoms (Aggarwal, 2013). According to the International Classification of Diseases, 10th Revision (ICD-10), acute hepatitis A is coded under B15–B15.9, acute hepatitis B under B16–B16.9, B17.0, B18.0–B18.1, B19.1–B19.11, acute hepatitis C under B17.1–B17.11, B18.2, B19.2–B19.21, and acute hepatitis E under B17.2. Chronic hepatitis B was defined by the presence of hepatitis B surface antigen (HBsAg) for more than 6 months (Lampertico et al., 2017), while chronic hepatitis C was confirmed by detecting both positive anti-HCV and positive HCV RNA at least 6 months after the initial infection (Kwo and John, 2019).

### 2.4 Statistical analysis

The age-standardized rates (ASRs) per 100,000 population for the incidence and prevalence of hepatitis were calculated using the following formula (Wang et al., 2021):


$$\begin{array}{l} \text{ASR }=\;\frac{\overset{A}{\underset{i=1}{\sum }}a_{i}w_{i}}{\overset{A}{\underset{i=1}{\sum }}w_{i}}\;\times 100,000 \end{array}$$

Where *a*_*i*_ denotes the *i*^*th*^ age subgroup and the number of persons (or weight) (*w*_*i*_) in the same age class *i* of the chosen reference standard population. The value was then divided by the sum of standard population weights. The ASRs were calculated using the age distribution weights of the reference population from GBD, which serves as the benchmark for all age groups (Schumacher et al., 2024). It is assumed that the natural logarithm of ASR changes is linear over time, represented by the equation *y* = α+β*x*+ε, where *y* = *ln*(*ASR*) and *x* is the calendar year and ε is the error term. The estimated annual percentage changes (EAPCs) in ASRs were calculated using the formula 100 × (exp(β)−1), with the corresponding 95% confidence interval (CI) derived from the linear regression model (Liu et al., 2019). If the EAPC and the lower bound of its 95% confidence interval (CI) were both positive, the ASR was considered to be on an upward trend. Conversely, if the EAPC estimate and the upper bound of its CI were negative, the ASR was deemed to be on a downward trend. The percentage change in a specific type of hepatitis between 1990 and 2021 was calculated as: (number of cases in 2021 – number of cases in 1990)/number of cases in 1990. Additionally, Pearson correlation analysis was conducted to evaluate the relationship between SDI levels and ASRs, and the results were visualized using Locally Weighted Scatterplot Smoothing (LOWESS) curves (Cleveland, 1979). All statistical analyses and mapping were performed using R software, version 4.1.0 (R Foundation for Statistical Computing), with a significance level set at *P* < 0.05 (2-tailed).

## 3 Results

### 3.1 Global viral hepatitis cases among WCBA

In 2021, it was estimated that globally there were 42,266,708 new cases and 109,107,759 prevalent cases of viral hepatitis among WCBA. This includes 19,519,113 new cases and 1,399,533 existing cases of AHA, 16,787,815 and 1,937,056 of AHB, 931,188 and 107,445 of AHC, 3,420,786 and 263,137 of AHE, 1,020,188 and 71,396,795 of CHB, and 587,617 and 34,003,793 of CHC (Supplementary Tables 2, 3). The age-standardized incidence rate (ASIR) per 100,000 population in 2021 was 1,012.3 (95% uncertainty interval [UI]: 1,010.9 to 1,013.7) for AHA, 856.7 (95% UI: 855.4 to 858) for AHB, 47.5 (95% UI: 47.2 to 47.8) for AHC, 178.2 (95% UI: 177.6 to 178.8) for AHE, 52.2 (95% UI: 51.9 to 52.5) for CHB, and 30 (95% UI: 29.8 to 30.2) for CHC (Supplementary Table 2). The age-standardized prevalence rate (ASPR) per 100,000 population in 2021 was 72.8 (95% UI: 72.4 to 73.2) for AHA, 98.8 (95% UI: 98.4 to 99.3) for AHB, 5.5 (95% UI: 5.4 to 5.6) for AHC, 13.7 (95% UI: 13.5 to 13.8) for AHE, 3,642.6 (95% UI: 3,634.2 to 3,651) for CHB, and 1,738.5 (95% UI: 1,732.7 to 1,744.3) for CHC (Supplementary Table 3).

Between 1990 and 2021, the global ASIR decreased annually by −1.11% (95% CI: −1.17 to −1.05) for AHA, −1.24% (95% CI: −1.33 to −1.15) for AHB, −0.18% (95% CI: −0.29 to −0.07) for AHC, −0.34% (95% CI: −0.38 to −0.29) for AHE, −1.33% (95% CI: −1.44 to −1.22) for CHB, and −0.29% (95% CI: −0.38 to −0.2) for CHC (Supplementary Table 2). Over the same period, the global ASPR increased annually by 0.46% (95% CI: 0.42 to 0.49) for AHA, while it decreased annually by −1.24% (95% CI: −1.33 to −1.15) for AHB, −0.18% (95% CI: −0.28 to −0.08) for AHC, −0.34% (95% CI: −0.38 to −0.29) for AHE, −1.22% (95% CI: −1.3 to −1.13) for CHB, and −0.85% (95% CI: −0.97 to −0.73) for CHC (Supplementary Table 3).

### 3.2 Regional viral hepatitis cases among WCBA

**Figure 1:**
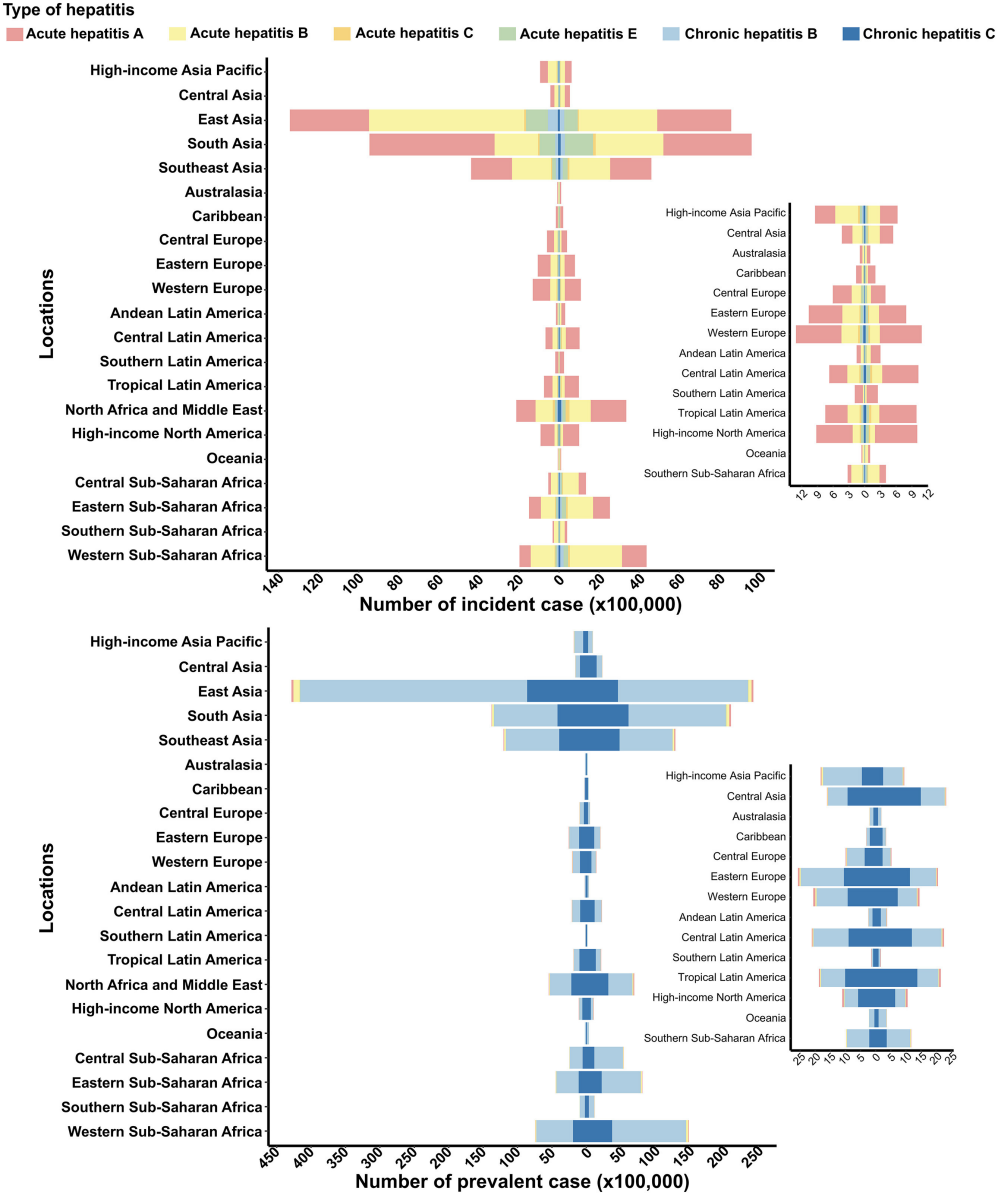
Incident and prevalent cases of viral hepatitis among women of childbearing age at the regional level in 1990 and 2021. The left column in each group represents data from 1990, while the right column represents data from 2021. Specific data from certain regions can be viewed on the right side of the panel.

The regions of East, South, and Southeast Asia reported the highest numbers of viral hepatitis incident and prevalent cases, followed by Sub-Saharan Africa and North Africa and the Middle East (Figure 1). The total number of viral hepatitis cases in these severely affected regions continued to rise from 1990 to 2021, with the exception of East Asia (Figure 1). The most prominent trend visible in the figure is the continued rise in viral hepatitis cases in South and Southeast Asia, Tropical Latin America, North Africa and Middle East, High-income North America, and regions of Sub-Saharan Africa (Figure 1). Among these regions, South Asia stands out for experiencing the largest decrease in acute hepatitis A (AHA) incidence cases, while simultaneously showing a notable increase in the prevalence of other type of viral hepatitis cases. Notably, South Asia saw the largest decrease in the number of AHA incidence cases and the greatest increase in prevalence cases (Supplementary Figures S1, S2).

**Figure 2:**
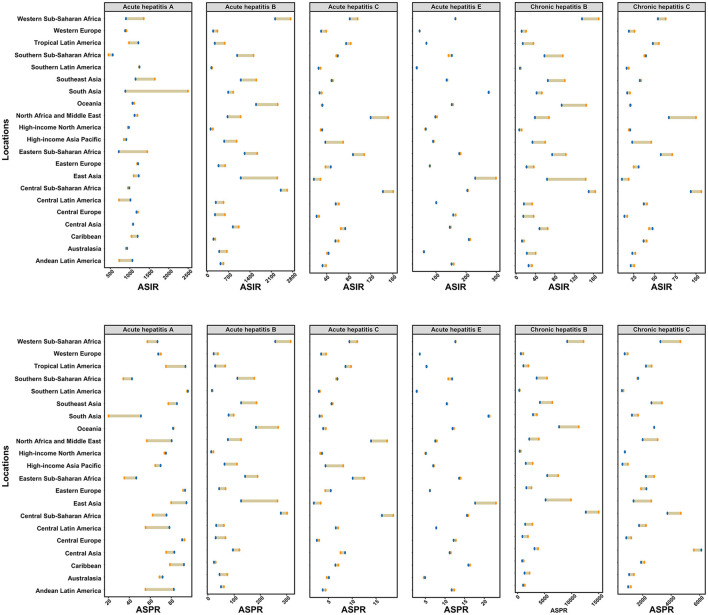
Age-standardized incidence and prevalence rates of six types of viral hepatitis among women of childbearing age across 21 GBD regions in 1990 and 2021. The yellow points represent data from 1990, while the blue points represent data from 2021. ASIR, age-standardized incidence rate; ASPR, age-standardized prevalence rate.

In 2021, Southern Latin America reported the highest ASIR and ASPR for AHA (Figure 2). Central Sub-Saharan Africa had the highest ASIR and ASPR for both acute and chronic hepatitis B, as well as the highest ASIR and ASPR for AHC. South Asia recorded the highest ASIR and ASPR for AHE. Meanwhile, Central Sub-Saharan Africa had the highest ASIR for CHC, while Central Asia reported the highest ASPR for CHC (Figure 2).

Between 1990 and 2021, the estimated annual percentage changes in ASIR and ASPR for specific types of viral hepatitis by location are shown in Supplementary Tables 2, 3. The fastest increase in ASIR for AHA was observed in Andean Latin America, with an annual change of 1.3% (95% CI: 1.24 to 1.35), while South Asia recorded the fastest increase in ASPR for AHA, with an annual change of 3.54% (95% CI: 3.31 to 3.77). Fortunately, all GBD regions showed a decreasing trend in the ASIR and ASPR for AHB and CHB. Eastern Europe experienced the fastest increase in ASIR for AHC and CHC, with annual changes of 0.86% (95% CI: 0.67 to 1.05) and 0.83% (95% CI: 0.64 to 1.03) per year, respectively. Similarly, Eastern Europe showed the fastest increase in ASPR for AHC and CHC, with annual changes of 0.87% (95% CI: 0.7 to 1.04) and 0.73% (95% CI: 0.44 to 1.01) per year, respectively. During the same period, the largest increases in ASIR and ASPR for AHE were recorded in Southern Sub-Saharan Africa, with an annual increase of 0.35%.

### 3.3 National viral hepatitis cases among WCBA

**Figure 3:**
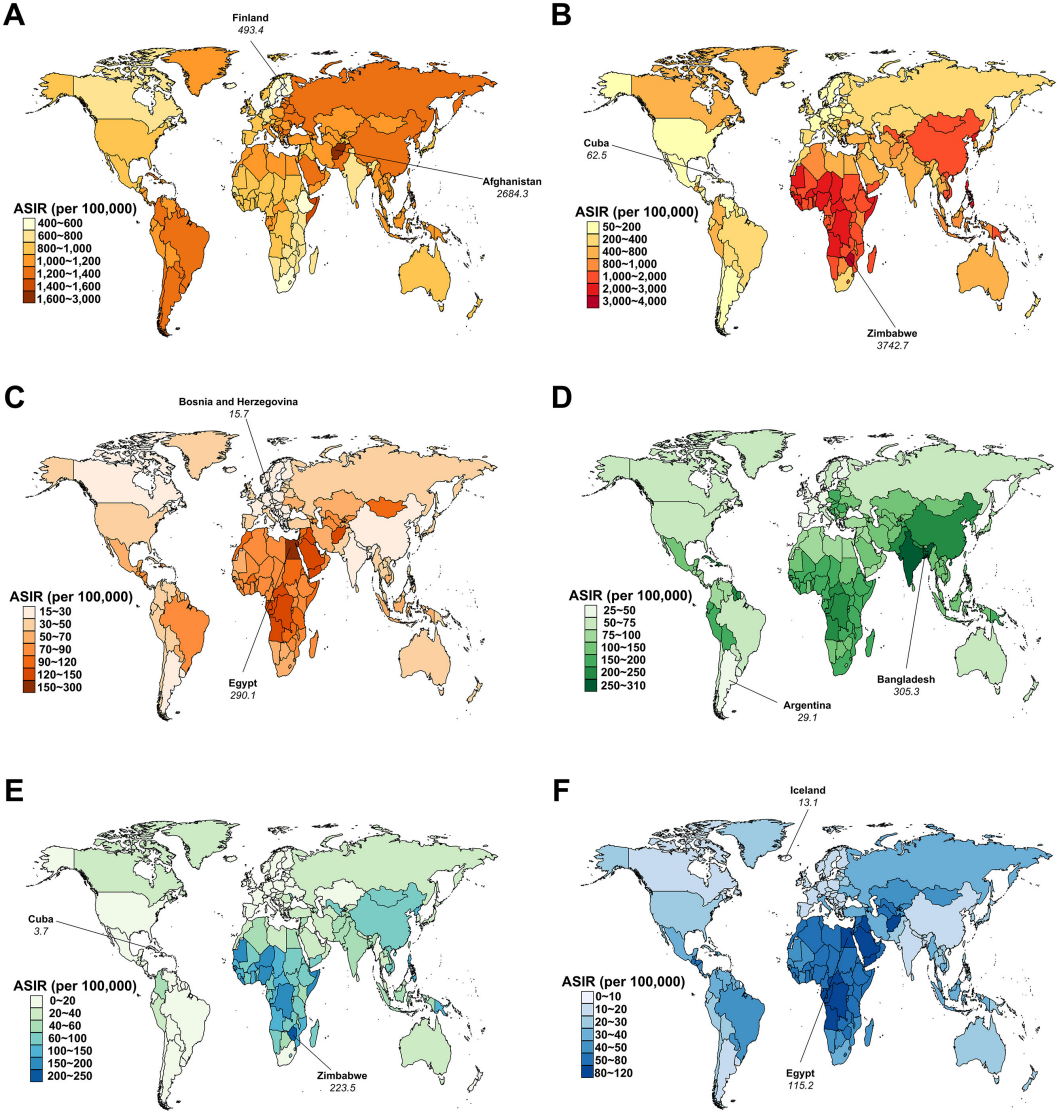
Age-standardized incidence rate (ASIR) for viral hepatitis among women of childbearing age across 204 countries and territories in 2021. **(A)** Acute hepatitis A; **(B)** Acute hepatitis B; **(C)** Acute hepatitis C; **(D)** Acute hepatitis E; **(E)** Chronic hepatitis B; **(F)** Chronic hepatitis C.

In 2021, Afghanistan had the highest ASIR for AHA at 2,684.3 (95% UI: 2,672.7 to 2,695.9) per 100,000 persons, while Argentina reported the highest ASPR at 97.7 (95% UI: 96.2 to 99.2) per 100,000 persons (Figure 3; Supplementary Figure S3). Zimbabwe had the highest ASIR and ASPR for AHB, at 3,742.7 (95% UI: 3,724.3 to 3,761.1) and 431.8 (95% UI: 426.0 to 437.6) per 100,000 persons, respectively. Egypt recorded the highest ASIR and ASPR for AHC, at 290.1 (95% UI: 288.0 to 292.2) and 33.5 (95% UI: 32.8 to 34.2) per 100,000 persons, respectively. Bangladesh had the highest ASIR and ASPR for AHE, at 305.3 (95% UI: 303.8 to 306.8) and 23.5 (95% UI: 23.1 to 23.9) per 100,000 persons, respectively. Zimbabwe also reported the highest ASIR for CHB at 223.5 (95% UI: 219.5 to 227.5) per 100,000 persons, while the Democratic Republic of the Congo had the highest ASPR for CHB at 14,239.3 (95% UI: 14,223.2 to 14,255.4) per 100,000 persons. Egypt had the highest ASIR for CHC at 115.2 (95% UI: 113.9 to 116.5) per 100,000 persons, while Mongolia recorded the highest ASPR for CHC at 9,483.6 (95% UI: 9,422.5 to 9,544.7) per 100,000 persons (Figure 3; Supplementary Figure S3).

**Figure 4:**
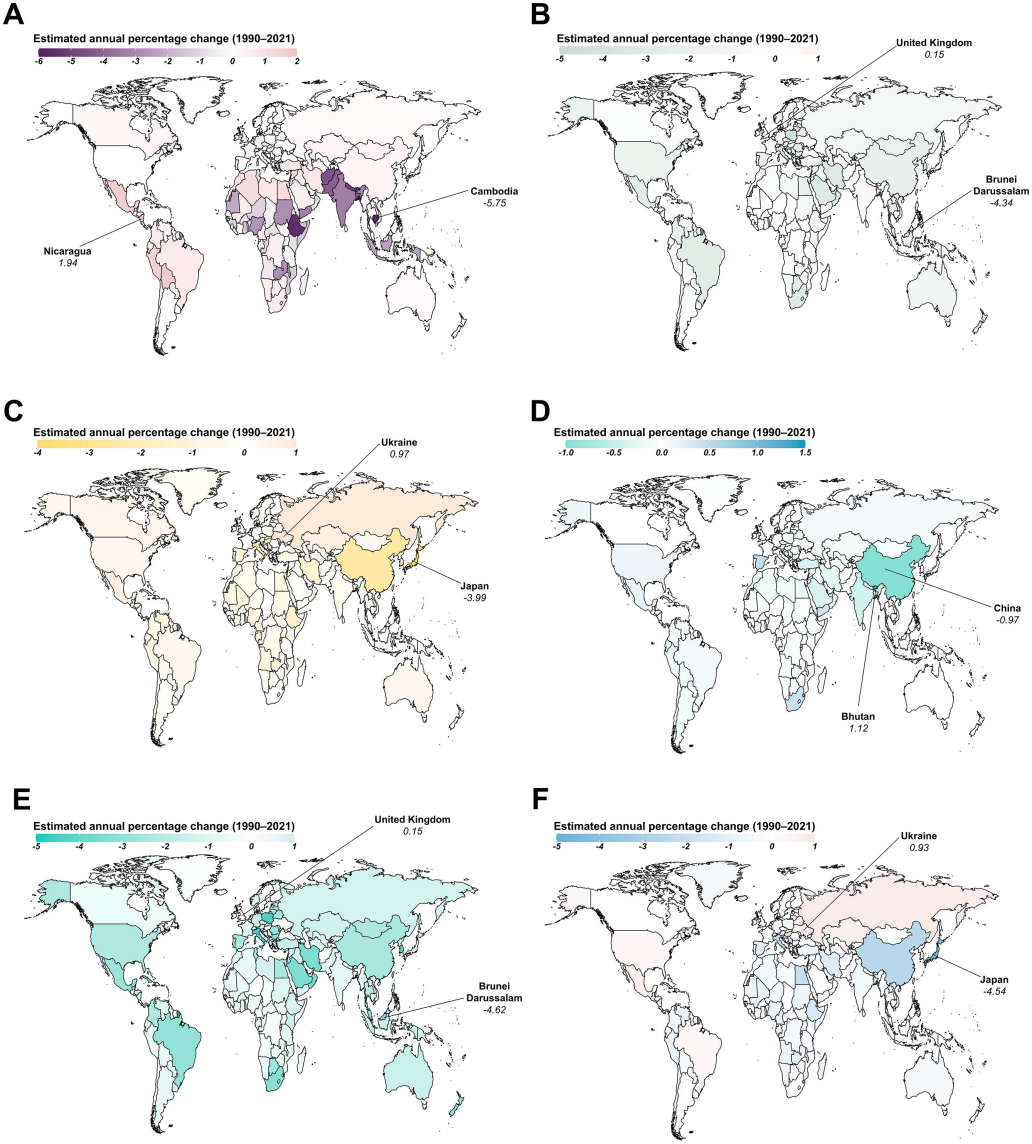
Estimated annual percentage changes in the age-standardized incidence rate (ASIR) for viral hepatitis among women of childbearing age across 204 countries and territories from 1990 to 2021. **(A)** Acute hepatitis A; **(B)** Acute hepatitis B; **(C)** Acute hepatitis C; **(D)** Acute hepatitis E; **(E)** Chronic hepatitis B; **(F)** Chronic hepatitis C.

From 1990 to 2021, Nicaragua exhibited the fastest increase in ASIR for AHA, with an annual change of 1.94% (95% CI: 1.74 to 2.15; Figure 4). However, the most significant increase in ASPR for AHA was observed in India, with an annual change of 3.87% (95% CI: 3.6 to 4.14; Supplementary Figure S4). During the same period, the United Kingdom saw the greatest increase in both ASIR and ASPR for AHB, with an annual change of 0.15% (Figure 4; Supplementary Figure S4). Similarly, Ukraine and Bhutan experienced the largest increases in both ASIR and ASPR for AHC and AHE, respectively, with annual changes of 0.97% for AHC and 1.12% for AHE. The fastest increase in ASIR for CHB was observed in the United Kingdom, with an annual change of 0.15% (95% CI: 0.11 to 0.18), while Denmark showed the fastest increase in ASPR for CHB, with an annual change of 0.25% (95% CI: 0.17 to 0.32). Ukraine experienced the greatest increases in both ASIR and ASPR for CHC, with annual changes of 0.93% (95% CI: 0.75 to 1.11) and 1.15% (95% CI: 0.8 to 1.51), respectively (Figure 4; Supplementary Figure S4).

### 3.4 Viral hepatitis cases among WCBA, by SDI

In 2021, both the ASIR and ASPR were significantly negatively associated with the SDI level for all types of viral hepatitis, except for AHA (Supplementary Figures S5, S6). Interestingly, the correlation between the ASPR of AHA and the SDI level followed an inverted “U” shape, peaking at SDI values around 0.69 before declining as SDI values increased (Supplementary Figure S6). Additionally, the contribution of low- and lower-middle-SDI regions to both the incidence and prevalence of viral hepatitis cases among WCBA has significantly increased over the past decades (Supplementary Figure S7).

Between 1990 and 2021, the proportion of AHA cases contributing to incident viral hepatitis among WCBA steadily increased in middle- and upper-SDI regions, while a growing contribution of AHB was observed in low-middle- and low-SDI regions (Supplementary Figure S8). In contrast, the proportions of most specific viral hepatitis types contributing to prevalent viral hepatitis cases remained stable across all five SDI quintiles during this period, with the exception of CHC, which exhibited an increasing trend in high-SDI regions (Supplementary Figure S9).

During the same period, the ASIR of AHA rose in high and high-middle SDI regions, while it decreased in regions with lower SDI levels. In low-SDI areas, the ASIR for AHA was surpassed by that for AHB (Supplementary Figure S10A). Meanwhile, the ASPR for CHB remained the highest among the six major types of viral hepatitis from 1990 to 2021. Notably, by 2021, the ASPR of CHB had decreased to a level close to that of CHC (Supplementary Figure S10B).

### 3.5 Age distribution of viral hepatitis cases among WCBA

Globally, the highest incidence and prevalence rates of AHA were observed in the 15–19 age group, with a notable increase in prevalence rates in regions with lower SDI levels within this age group from 1990 to 2021 (Supplementary Figures S11, S12). For AHB, the highest incidence and prevalence rates were observed in women aged 25 to 29 years, while individuals aged 15–19 years experienced the most rapid decrease. Both incidence and prevalence rates for AHC and CHC rise with age, whereas the rates for AHE decrease with increasing age (Supplementary Figures S11, S12). In 1990, younger WCBA had higher rates of CHB compared to older women. However, the 15–19 age group experienced the fastest decline over the past decades, reaching the lowest rates by 2021 (Supplementary Figures S11, S12).

**Figure 5:**
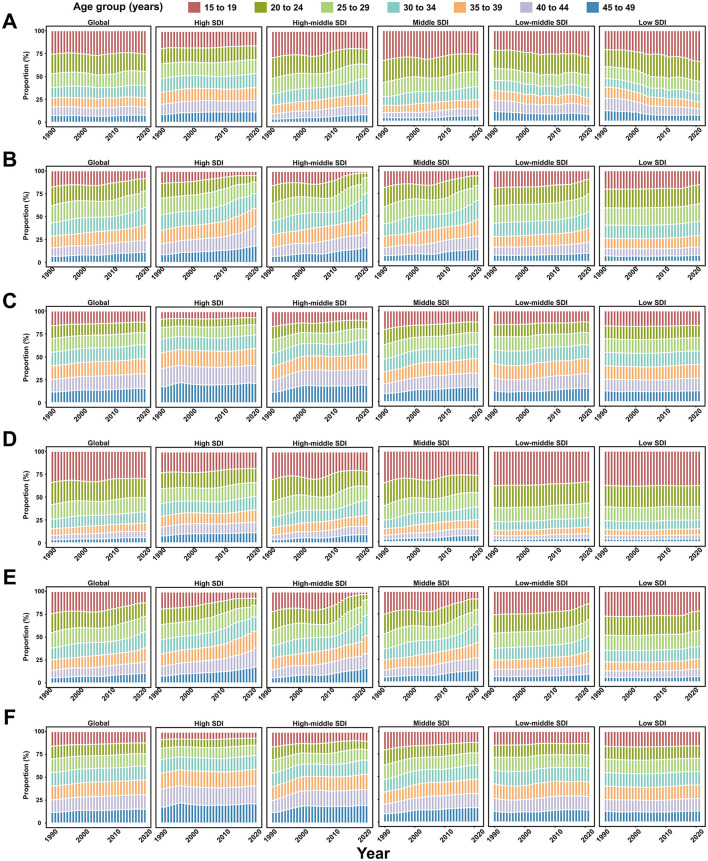
Seven age groups as percentages of the total incident cases of viral hepatitis among women of childbearing age globally, and in territories with low to high SDIs, between 1990 and 2021. **(A)** Acute hepatitis A; **(B)** Acute hepatitis B; **(C)** Acute hepatitis C; **(D)** Acute hepatitis E; **(E)** Chronic hepatitis B; **(F)** Chronic hepatitis C.

Between 1990 and 2021, the proportion of younger age groups contributing to the number of incident and prevalent viral hepatitis cases remained stable or showed a declining trend (Figure 5; Supplementary Figure S13). However, younger WCBA increasingly accounted for incident AHA cases in low-middle- and low-SDI regions (Figure 5). Additionally, the contribution of younger age groups to the number of incident and prevalent viral hepatitis cases decreased as the SDI level increased (Figure 5; Supplementary Figure S13).

**Figure 6:**
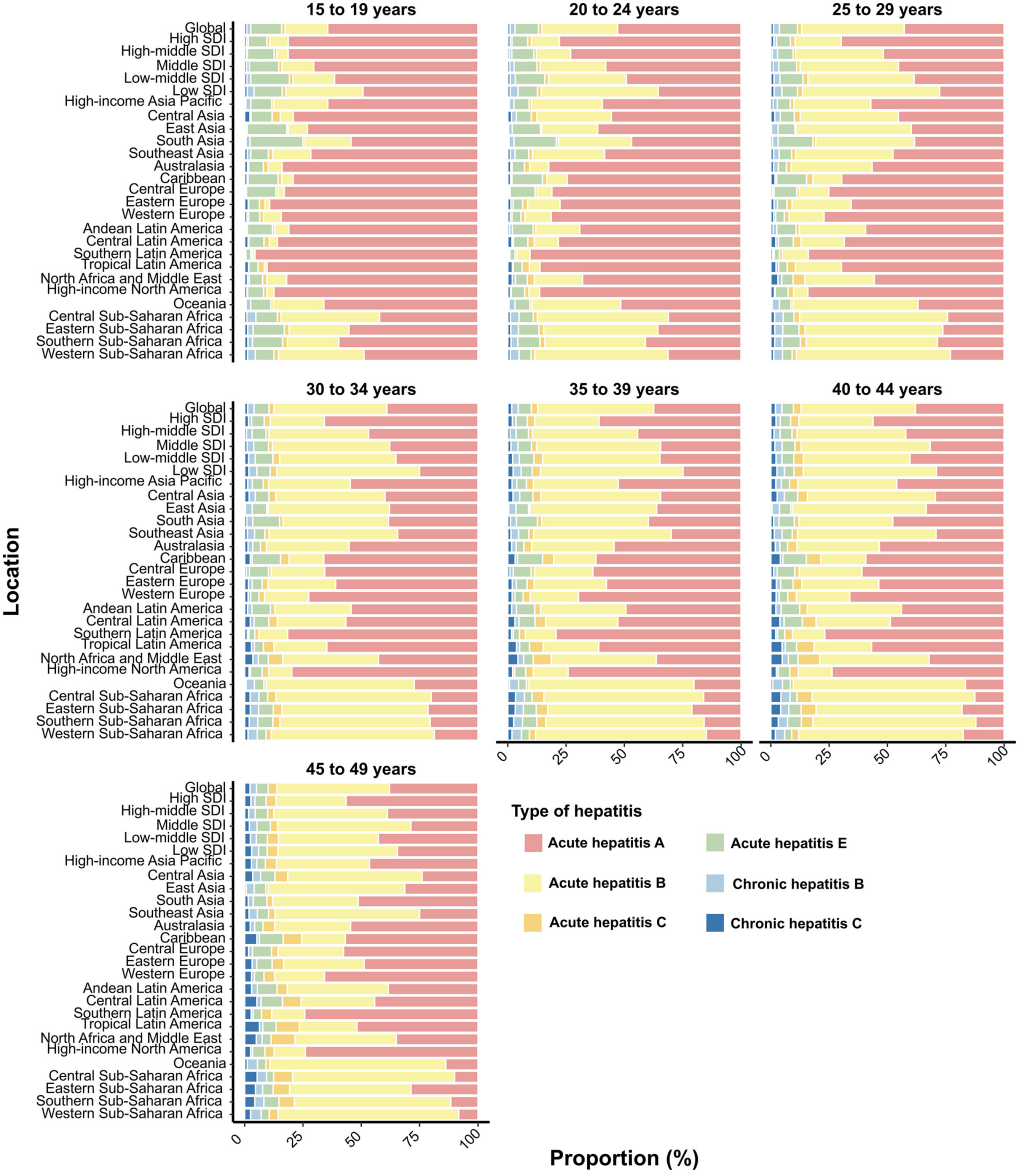
Contribution of six types of viral hepatitis to incident cases among women of childbearing age globally, in territories with low to high SDIs, and across 21 GBD regions in 2021, categorized by age group. SDI, Socio-Demographic Index.

In 2021, the proportion of AHA contributing to incident viral hepatitis cases decreased with increasing age globally, across territories with low to high SDIs, and in the 21 GBD regions, while AHB showed an opposite trend (Figure 6). Notably, in Sub-Saharan Africa, AHB contributed more to incident viral hepatitis cases than AHA among WCBA, except for those aged 15–19 years (Figure 6). By age group, CHB was the most prevalent form of viral hepatitis in Asian regions (excluding Central Asia) and Oceania, with its proportion increasing with age (Supplementary Figure S14). However, in most of Europe, the Americas, and Central Asia, CHC was the primary contributor to prevalent viral hepatitis cases among WCBA (Supplementary Figure S14).

## 4 Discussion

Women of childbearing age represent nearly a quarter of the global population, and their health is closely linked to the wellbeing of the next generation (Dugan et al., 2021). WCBA are considered a high-risk population for hepatitis due to several factors, including the potential for vertical transmission, the impact of hepatitis on pregnancy outcomes, and the overall burden of the disease in this demographic (Medicine, 2023). Nevertheless, the assessment of the burden of viral hepatitis among all women of reproductive age (15–49 years) is often underrepresented in previous studies. To the best of our knowledge, this is the first study to evaluate the incidence and prevalence rates of viral hepatitis among individuals of reproductive age from 1990 to 2021 at global, regional, and national levels. In 2021, an estimated 109 million WCBA were living with viral hepatitis worldwide, with the majority of cases due to CHB. This burden is particularly concentrated in East and South Asia, where it has continued to grow over the past decades. This trend aligns with the WHO's call for equitable access to viral hepatitis interventions in low- and middle-income countries (World Health Organization, 2024). Our study provides a comprehensive analysis of viral hepatitis burden among women of childbearing age (WCBA) from 1990 to 2021, representing a significant addition to the literature, as few studies have examined this demographic in detail. Our findings reveal a concentrated burden of hepatitis, particularly CHB, in East and South Asia, which aligns with the broader trends observed in previous studies, such as the GBD 2019 estimates. Notably, the GBD 2019 report showed a steady decline in global hepatitis B incidence, largely driven by the expansion of vaccination programs (Sheena et al., 2022). However, our study highlights that while HBV incidence decreased in higher-income regions, low-SDI regions like Sub-Saharan Africa continue to experience an increase in the absolute number of HBV cases, a trend that the GBD 2019 also noted but did not focus on in women of reproductive age specifically. Furthermore, our findings point to emerging patterns of hepatitis A in regions like Latin America and the Caribbean, which contrast with the trend observed in the Asia Pacific region where the GBD 2019 identified a growing burden among older adults due to delayed vaccination (Zeng et al., 2021). These differences underline the dynamic nature of hepatitis epidemiology and emphasize the need for tailored public health strategies to address emerging trends in specific populations, including WCBA.

A previous study highlighted that the most significant increases in the ASIR of AHA for both sexes across all age groups were observed in the high-income Asia Pacific region and Australasia (Cao et al., 2021). This trend is likely driven by the rising number of cases among older adults aged 60 and above in these regions. In high-income countries, limited vaccination coverage against the hepatitis A virus (HAV) among adults has left a substantial portion of the older population vulnerable to infection (Jacobsen and Wiersma, 2010; Ly and Klevens, 2015). However, our study identified that the most rapid increase in the ASIR of AHA among WCBA occurred in Latin America and the Caribbean. As socioeconomic conditions improve and childhood exposure decreases, the Age at Midpoint of Population Immunity (AMPI)—the youngest age at which half of a birth cohort exhibits serologic evidence of prior HAV exposure—is shifting to adolescence and early adulthood in many middle-income regions (Lemon et al., 2018; Van Effelterre et al., 2017). Mathematical models from Brazil and Mexico suggest that as the overall incidence of HAV declines, the proportion of symptomatic cases among young adults rises (Van Effelterre et al., 2017). This epidemiological shift reflects changing vaccination policies and improved sanitation that have led to lower childhood exposure to HAV. As these populations age, the incidence of hepatitis A among adults, particularly those in high-risk groups like WCBA, has risen. In Latin America, the increasing burden of hepatitis A highlights the need for a re-evaluation of vaccination strategies, particularly considering expanding vaccination coverage to protect adolescents and adults who are now at increased risk due to their lack of prior exposure. Similarly, our findings indicate that the contribution of younger age groups to the incidence of AHA has increased in low-middle- and low-SDI regions while decreasing in regions with higher SDI levels. Consequently, changes in the epidemiological patterns can disproportionately affect specific demographics, including WCBA (Parellada et al., 2022). More importantly, consistent with previous studies (Xiao et al., 2024; Cao et al., 2021), the incidence of AHA among WCBA is decreasing in hyper-endemic regions, while it is rising in low-endemic regions. Understanding these complex and evolving epidemiological patterns is crucial for developing effective interventions. In low-endemic regions where AHA is emerging, vaccination efforts typically target high-risk populations, such as injection drug users and international travelers (Wasley et al., 2006). However, an immediate re-evaluation of vaccine strategies may be needed, including expanding vaccination to protect adolescents and adults who remain susceptible to infection (Wang et al., 2016). Meanwhile, the escalating burden of AHA among WCBA in Sub-Saharan Africa demands urgent attention, particularly given the region's rapidly increasing population and high fertility rates (Wang et al., 2020). To prevent future outbreaks, it is essential to focus on public health education that emphasizes the importance of vaccination, along with ensuring access to clean water and proper waste disposal (Aggarwal and Goel, 2015). Many countries, especially those in regions with high hepatitis A burden (e.g., parts of Africa and South Asia), have introduced hepatitis A vaccination campaigns. For example, India has launched national programs targeting children to curb outbreaks (Verma and Khanna, 2012).

The incidence and prevalence of both acute and chronic hepatitis B among WCBA declined across all GBD regions, primarily due to increased HBV vaccination rates (Zeng et al., 2021). This trend corresponds with the more rapid reductions in HBV prevalence among children and infants compared to all age groups since 1990 (Sheena et al., 2022). However, in low-SDI regions, such as Sub-Saharan Africa, the total number of hepatitis B cases rose from 1990 to 2021, with HBV accounting for more than half of the new cases of viral hepatitis among WCBA. Worryingly, only about 6% of neonates had received timely HBV birth-dose vaccine in the African region in 2019 (Noubiap and Ndoula, 2022). Due to gaps in antenatal screening, limited access to the HBV birth-dose vaccination, and inconsistent availability of antiviral prophylaxis, MTCT of HBV remains a major mode of transmission in the African region, which accounts for over 90% of new infections in children worldwide (Nartey and Bockarie, 2024). *Thompson and colleagues* have demonstrated that integrating HBV testing and treatment, as well as providing the monovalent HBV birth-dose vaccine as part of routine maternal and child health services, is both feasible and acceptable (Thompson et al., 2021). This strategy could significantly accelerate efforts toward eliminating HBV in Africa.

In Asia, the implementation of the hepatitis B birth dose vaccination has shown varying levels of success, particularly in countries with high-endemicity such as China, Vietnam, India, and Indonesia (Al-Busafi and Alwassief, 2024). In contrast, European countries have been leaders in hepatitis B vaccination programs for many years, with birth dose vaccination seamlessly integrated into their national schedules (Lernout et al., 2014). These countries face fewer challenges related to vaccine access and cold chain management, as their healthcare systems are generally well-established, and there is widespread public awareness of hepatitis B. In the Middle East and North Africa, the picture is more mixed. Countries like Saudi Arabia, the United Arab Emirates, and Qatar have implemented universal hepatitis B vaccination programs, ensuring that all newborns receive the birth dose within the first 24 h (Akyildiz et al., 2020). However, Egypt and Algeria, which began birth dose vaccination in the 1990s, still face challenges in achieving full coverage, particularly in rural or underserved regions (Salama et al., 2015). In Latin America, most countries have successfully incorporated the hepatitis B birth dose into their routine immunization schedules, leading to significant progress in reducing HBV transmission (Velandia-González et al., 2021). Nevertheless, some countries in the Caribbean still encounter barriers to implementation, particularly in remote islands where access to vaccines remains limited. In summary, while the hepatitis B birth dose is a crucial intervention in preventing mother-to-child transmission of HBV, its implementation varies significantly across regions. Enhanced international cooperation, supported by organizations such as Gavi, the World Health Organization, and UNICEF, is essential to accelerating the adoption and expansion of hepatitis B vaccination programs, particularly in high-burden regions (Cui, 2020).

Moreover, vaccination alone is insufficient to eliminate the burden of HBV infection in high-endemic regions (Noubiap and Ndoula, 2022). A large meta-analysis estimated that, as of 2021, approximately 43.3 million people in China are still chronically infected with HBV, even three decades after the introduction of infant HBV vaccination (Zheng et al., 2018; Liu et al., 2023). Alarmingly, the prevalence of HBV infection remains relatively high among WCBA in China, with nearly 18.0 million affected in 2021. In this study, we also analyzed data from pregnant women screened at the obstetric outpatient clinics of Jiangmen Maternity and Child Health Care Hospital, the largest maternity and child specialist hospital in Jiangmen city, from 2017 to 2023 (Supplementary Figure S15). As shown in Supplementary Table S4, while the prevalence of HBV infection among pregnant women decreased significantly from 2017 to 2023, it remained high, with over 5% of pregnant women still affected by HBV infection in 2023. Therefore, more comprehensive diagnosis and treatment efforts are urgently needed to reduce the global burden of HBV (Revill et al., 2016; Liu et al., 2024). Unexpectedly, among the 204 countries and territories surveyed, Denmark and the UK have shown an increasing trend in the burden of hepatitis B among WCBA. This rise is primarily attributed to high-risk groups, such as immigrants from regions with high HBV prevalence, individuals with multiple sexual partners, and those with a history of injection drug use (Bivegete et al., 2023). For instance, the prevalence of HBsAg-positive mothers in Denmark more than doubled between 1965 and 2005, although it decreased 10-fold among women of Danish origin (Harder et al., 2011). Reportedly, about 0.4% of pregnant women in England and 0.26% in Denmark have HBV infection (Harder et al., 2011; Bailey et al., 2023). In comparison, our study found that approximately 0.9% of WCBA in the UK and 0.7% in Denmark are living with chronic hepatitis B. Differences in prevalence from earlier surveys may be due to variations in the age groups of pregnant women vs. women of reproductive age. However, age alone is not the only contributing factor, and the genetic diversity of HBV plays a crucial role in its prevalence and transmission dynamics (Revill et al., 2020; Liu et al., 2018). HBV genotypic variations and intergenotypic recombination on HBV epidemiology, particularly in relation to different populations such as pregnant women and women of reproductive age.

We found that approximately 1.7% of WCBA worldwide might be living with HCV in 2021, which is more than twice the estimate by Dugan et al. (2021), who reported an HCV viremic prevalence of 0.78% among this population in 2019. This disparity may be due to variations in models and data quality between the two datasets. For instance, datasets from developed countries tend to be more comprehensive, thanks to better perinatal HCV screening programs, compared to those from many low-income countries (Abbasi et al., 2023). As a result, the GBD may have assigned greater weight to data from high-income countries than to data from underdeveloped countries (Ferrari et al., 2024). Additionally, the incidence rate of hepatitis C increased significantly across Eastern Europe, Central Asia, and high-income North America, where people who inject drugs are the primary source of HCV infection (Degenhardt et al., 2023). For example, Ukraine had the highest prevalence of HCV among children and teenagers in Eastern Europe, and the rate of illicit drug use among teenagers in Ukraine has risen from 12% to 18% (Isakov et al., 2021). Similarly, numerous studies in the United States have reported rising trends HCV incidence among WCBA, coinciding with the ongoing opioid epidemic (Kushner and Reau, 2021). Despite significant advancements in hepatitis C treatment through the development of direct-acting antivirals, there is still no approved treatment for HCV infection during pregnancy (Kushner et al., 2018). Therefore, universal preconception testing and treatment for WCBA is an effective strategy to identify those with HCV, helping to protect the health of both mothers and their children (Judd et al., 2021). In 1990, Egypt had the second-highest prevalence of HCV infection among WCBA worldwide. However, with the implementation of community-based mass HCV screening and treatment programs (Waked et al., 2020), Egypt significantly reduced its HCV prevalence and ranked 46th in terms of HCV impact by 2021. Despite this progress, current data indicates that HCV transmission continues in Egypt, with the highest incidence rate among WCBA compared to other countries (Shiha et al., 2020). From 1990 to 2021, Mongolia, Uzbekistan, Kyrgyzstan, and Kazakhstan experienced the highest prevalence of HCV among WCBA. Molecular clock studies indicate that the HCV outbreak in Mongolia began due to unsafe healthcare practices (Chaabna et al., 2021). Therefore, prioritizing the prevention of HCV transmission in healthcare settings is crucial in resource-limited countries (Botheju et al., 2019).

HEV infection sharing similar endemic regions with HAV as a waterborne or foodborne infection, and is highly prevalent in East Asia, South Asia, and Sub-Saharan Africa (The Lancet Gastroenterology and Hepatology, 2016). The global ASIR of AHE among WCBA has remained relatively stable, showing a slight decline in most GBD regions, but a more significant decrease in East Asia. In China, improvements in water supply and sanitation systems over the past few decades have led to a shift in HEV transmission from waterborne to foodborne routes (Sridhar et al., 2017). Conversely, an unfavorable trend in the incidence of acute hepatitis E (AHE) has been observed in Southern Sub-Saharan Africa, largely associated with refugee and Internally Displaced Persons (IDPs) camps (Bagulo et al., 2020). Individuals experiencing homelessness are particularly vulnerable to infections due to limited access to safe housing, clean water, vaccinations, healthcare, and the psychological challenges stemming from social marginalization (Liang et al., 2023). While there is currently no widely available vaccine for hepatitis E, China has developed a hepatitis E vaccine (HEV239), which was approved for use in 2011 (Li et al., 2015). The vaccine has shown effectiveness in preventing HEV infection, particularly in high-risk populations, such as pregnant women and individuals living in endemic areas (Zhang et al., 2015; Marti et al., 2024). However, its use is currently limited to China, where it has been incorporated into vaccination programs in areas with high HEV burden. The safety and effectiveness of the Hepatitis E vaccine (recombinant HEV vaccine HEV239) in WCBA in rural Bangladesh have been studied (Zaman et al., 2024), and the WHO has determined that the benefits of vaccination outweigh the risks in fragile, conflict-affected, and vulnerable settings where hepatitis E virus circulation is documented (Marti et al., 2024). Consequently, widespread Hepatitis E vaccination of women of child-bearing age would provide continued benefits, particularly for pregnant women in these vulnerable environments (Marti et al., 2024). Additionally, HEV infection remains an emerging concern in high-SDI regions, such as High-income North America and Australasia. These areas implemented water control policies earlier and have achieved stable levels of disease management, resulting in significantly lower incidence rates. However, recent epidemiological data indicate an increasing seroprevalence of anti-HEV antibodies in industrialized countries, raising concerns about the safety of blood product transfusions (Pawlotsky, 2014; King et al., 2018).

The GBD 2021 report does not include data on hepatitis D; however, it is important to highlight countries where hepatitis B subjects are known to be co-infected with hepatitis D, as this co-infection can significantly impact disease progression and treatment outcomes (Stockdale et al., 2020; Negro, 2014). While precise global data on hepatitis B and D co-infection remains limited, several countries, particularly in regions with a high burden of hepatitis B, have reported notable co-infection rates (Stockdale et al., 2020). For example, in Sub-Saharan Africa, Eastern Europe, and South Asia, where chronic hepatitis B is more prevalent, co-infection with hepatitis D has been documented in a significant proportion of patients with chronic hepatitis B (Stockdale et al., 2017). Countries such as Turkey, Italy, Egypt, and Vietnam have identified hepatitis B patients who are co-infected with hepatitis D, contributing to the complexity of managing these individuals due to the more aggressive course of the disease.

This study had several limitations. Firstly, the data were uneven, particularly in low-income countries, which are estimated to have a high disease burden but lack sufficient data sources. For years and locations where data are missing, GBD estimates rely on predictive covariates, regional levels, and consistent temporal trends, which can affect the accuracy and reliability of viral hepatitis case estimates due to methodological issues in the GBD Study (Ferrari et al., 2024). While the GBD model provides a comprehensive global view of disease burden, its reliance on estimates and model-based projections introduces limitations, particularly regarding regions with limited or unreliable data. Second, the burden of Hepatitis D, which is not rare but often underdiagnosed (Stockdale et al., 2020), is not covered in GBD 2021. Third, our study was not able to analyze the burden of viral hepatitis among WCBA by viral genotype or sub-genotype. These genotypes and sub-genotypes have distinct geographical distributions and can significantly impact the management and control of the infection (Lu et al., 2006; Tian and Jia, 2016; Gower et al., 2014; Robertson et al., 1992). Despite these limitations, our study provides current information that will support the development of targeted public health strategies to reduce global viral hepatitis burden.

## 5 Conclusions

Viral hepatitis among WCBA represents a significant global public health challenge. While the global incidence of viral hepatitis has declined between 1990 and 2021, many regions have seen an increase in the absolute number of cases. Notably, Sub-Saharan Africa and North Africa/Middle East have witnessed substantial rises in both incident and prevalent cases. Regions with low to middle SDI are particularly affected, with younger women being especially vulnerable. To address this, targeted vaccination programs for hepatitis B and A should be incorporated into maternal and child health services, ensuring WCBA have access to preventive care before, during, and after pregnancy. Additionally, widespread prenatal screening for viral hepatitis is essential to identify and manage chronic hepatitis B and C cases.

## Data Availability

The original contributions presented in the study are included in the article/Supplementary material, further inquiries can be directed to the corresponding author.

## Supplementary material

Supplementary Figure S1 Percent changes in incident viral hepatitis cases among women of childbearing age between 1990 and 2021, globally and across 21 GBD regions. **(A)** Acute hepatitis A; **(B)** Acute hepatitis B; **(C)** Acute hepatitis C; **(D)** Acute hepatitis E; **(E)** Chronic hepatitis B; **(F)** Chronic hepatitis C.

Supplementary Figure S2 Percent changes in prevalent viral hepatitis cases among women of childbearing age between 1990 and 2021, globally and across 21 GBD regions. **(A)** Acute hepatitis A; **(B)** Acute hepatitis B; **(C)** Acute hepatitis C; **(D)** Acute hepatitis E; **(E)** Chronic hepatitis B; **(F)** Chronic hepatitis C.

Supplementary Figure S3 Age-standardized prevalence rate (ASPR) for viral hepatitis among women of childbearing age across 204 countries and territories in 2021. **(A)** Acute hepatitis A; **(B)** Acute hepatitis B; **(C)** Acute hepatitis C; **(D)** Acute hepatitis E; **(E)** Chronic hepatitis B; **(F)** Chronic hepatitis C.

Supplementary Figure S4 Estimated annual percentage changes in the age-standardized prevalence rate (ASPR) for viral hepatitis among women of childbearing age across 204 countries and territories from 1990 to 2021. **(A)** Acute hepatitis A; **(B)** Acute hepatitis B; **(C)** Acute hepatitis C; **(D)** Acute hepatitis E; **(E)** Chronic hepatitis B; **(F)** Chronic hepatitis C.

Supplementary Figure S5 Correlation between the age-standardized incidence rate (ASIR) of viral hepatitis among women of childbearing age and SDI levels in 2021. **(A)** Acute hepatitis A; **(B)** Acute hepatitis B; **(C)** Acute hepatitis C; **(D)** Acute hepatitis E; **(E)** Chronic hepatitis B; **(F)** Chronic hepatitis C. SDI, Sociodemographic index.

Supplementary Figure S6 Correlation between the age-standardized prevalence rate (ASPR) of viral hepatitis among women of childbearing age and SDI levels in 2021. **(A)** Acute hepatitis A; **(B)** Acute hepatitis B; **(C)** Acute hepatitis C; **(D)** Acute hepatitis E; **(E)** Chronic hepatitis B; **(F)** Chronic hepatitis C. SDI, Sociodemographic index.

Supplementary Figure S7 The contributions of the five SDI regions to the incidence and prevalence of specific viral hepatitis cases among women of childbearing age from 1990 to 2021. **(A)** Incident viral hepatitis cases; **(B)** Prevalent viral hepatitis cases. SDI, Sociodemographic Index.

Supplementary Figure S8 The six types of viral hepatitis as percentages of total incident cases among women of childbearing age globally, in territories with low to high SDIs, and across 21 GBD regions between 1990 and 2021. SDI, Socio-Demographic Index.

Supplementary Figure S9 The six types of viral hepatitis as percentages of total prevalent cases among women of childbearing age globally, in territories with low to high SDIs, and across 21 GBD regions between 1990 and 2021. SDI, Socio-Demographic Index.

Supplementary Figure S10 Age-standardized **(A)** incidence and **(B)** prevalence rates of viral hepatitis among women of childbearing age per 100,000 people from 1990 to 2021 worldwide and across SDI regions. SDI, Sociodemographic index.

Supplementary Figure S11 Age-specific incidence rate of viral hepatitis among women of childbearing age globally and across five SDI regions from 1990 to 2021. **(A)** Acute hepatitis A; **(B)** Acute hepatitis B; **(C)** Acute hepatitis C; **(D)** Acute hepatitis E; **(E)** Chronic hepatitis B; **(F)** Chronic hepatitis C. SDI, Sociodemographic index.

Supplementary Figure S12 Age-specific prevalence rate of viral hepatitis among women of childbearing age globally and across five SDI regions from 1990 to 2021. **(A)** Acute hepatitis A; **(B)** Acute hepatitis B; **(C)** Acute hepatitis C; **(D)** Acute hepatitis E; **(E)** Chronic hepatitis B; **(F)** Chronic hepatitis C. SDI, Sociodemographic index.

Supplementary Figure S13 The seven age groups as percentages of total prevalent viral hepatitis cases among women of childbearing age globally and in territories with low to high SDIs between 1990 and 2021. SDI, Sociodemographic index.

Supplementary Figure S14 Contribution of six types of viral hepatitis to prevalent cases among women of childbearing age globally, in territories with low to high SDIs, and across 21 GBD regions in 2021, categorized by age group. SDI, Socio-Demographic Index.

Supplementary Figure S15 The number of pregnant women screened for hepatitis B infection at the obstetric outpatient clinics of Jiangmen Maternal and Child Care Hospital in China, from 2017 to 2023.

Supplementary Table 1 21 GBD world regions and 204 countries and territories within each region.

Supplementary Table 2 Global incidence of hepatitis in women aged 15–49 years in 1990 and 2021, and estimated annual percentage changes from 1990 to 2021.

Supplementary Table 3 Global prevalence of hepatitis in women aged 15–49 years in 1990 and 2021, and estimated annual percentage changes from 1990 to 2021.

Supplementary Table 4 Prevalence of hepatitis B infection among pregnant women screened at the obstetric outpatient clinics of Jiangmen Maternal and Child Care Hospital in China, 2017 to 2023.
