# Supplementary material for: Global burden and trends of viral hepatitis among women of childbearing age from 1990 to 2021
Source: Front Microbiol. 2025 Feb 21;16:1553129. doi: 10.3389/fmicb.2025.1553129 (PMC11885505; doi:10.3389/fmicb.2025.1553129)

Supplementary Table 1. 21 GBD world regions and 204 countries and territories within each region.

GBD World Region (n=21)	Countries and territories (n=204)
Central Asia	Armenia, Azerbaijan, Georgia, Kazakhstan, Kyrgyzstan, Mongolia, Tajikistan, Turkmenistan, Uzbekistan
Central Europe	Albania, Bosnia and Herzegovina, Bulgaria, Croatia, Czech Republic, Hungary, Montenegro, North Macedonia, Poland (subnational), Romania, Serbia, Slovakia, Slovenia
Eastern Europe	Belarus, Estonia, Latvia, Lithuania, Moldova, Russia (subnational), Ukraine
Australasia	Australia, New Zealand (subnational Māori + non-Māori)
High-income Asia Pacific	Brunei, Japan (subnational), Singapore, South Korea
High-income North America	Canada, Greenland, United States (subnational)
Southern Latin America	Argentina, Chile, Uruguay
Western Europe	Andorra, Austria, Belgium, Cyprus, Denmark, Finland, France, Germany, Greece, Iceland, Ireland, Israel, Italy (subnational), Luxembourg, Malta, Monaco, Netherlands, Norway (subnational), Portugal, San Marino, Spain, Sweden (subnational), Switzerland, United Kingdom (subnational)
Andean Latin America	Bolivia, Ecuador, Peru
Caribbean	Antigua and Barbuda, Bahamas, Barbados, Belize, Bermuda, Cuba, Dominica, Dominican Republic, Grenada, Guyana, Haiti, Jamaica, Puerto Rico, Saint Kitts and Nevis, Saint Lucia, Saint Vincent and the Grenadines, Suriname, Trinidad and Tobago, US Virgin Islands
Central Latin America	Colombia, Costa Rica, El Salvador, Guatemala, Honduras, Mexico (subnational), Nicaragua, Panama, Venezuela
Tropical Latin America	Brazil (subnational), Paraguay
North Africa and Middle East	Afghanistan, Algeria, Bahrain, Egypt, Iran (subnational), Iraq, Jordan, Kuwait, Lebanon, Libya, Morocco, Oman, Palestine, Qatar, Saudi Arabia, Sudan, Syria, Tunisia, Türkiye, United Arab Emirates, Yemen
South Asia	Bangladesh, Bhutan, India (subnational), Nepal, Pakistan (subnational)

East Asia	China, North Korea, Taiwan (province of China)
Oceania	American Samoa, Cook Islands, Federated States of Micronesia, Fiji, Guam, Kiribati, Marshall Islands, Nauru, Niue, Northern Mariana Islands, Palau, Papua New Guinea, Samoa, Solomon Islands, Tokelau, Tonga, Tuvalu, Vanuatu
Southeast Asia	Cambodia, Indonesia (subnational), Laos, Malaysia, Maldives, Mauritius, Myanmar, Philippines (subnational), Seychelles, Sri Lanka, Thailand, Timor-Leste, Vietnam
Central sub-Saharan Africa	Angola, Central African Republic, Congo (Brazzaville), Democratic Republic of the Congo, Equatorial Guinea, Gabon
Eastern sub-Saharan Africa	Burundi, Comoros, Djibouti, Eritrea, Ethiopia (subnational), Kenya (subnational), Madagascar, Malawi, Mozambique, Rwanda, Somalia, South Sudan, Tanzania, Uganda, Zambia
Southern sub-Saharan Africa	Botswana, eSwatini, Lesotho, Namibia, South Africa (subnational), Zimbabwe
Western sub-Saharan Africa	Benin, Burkina Faso, Cape Verde, Cameroon, Chad, Côte d’Ivoire, Gambia, Ghana, Guinea, Guinea-Bissau, Liberia, Mali, Mauritania, Niger, Nigeria (subnational), São Tomé and Príncipe, Senegal, Sierra Leone, Togo

Supplementary Table 2. Global incidence of hepatitis in women aged 15–49 years in 1990 and 2021, and estimated annual percentage changes from 1990 to 2021

Characteristics	1990		2021		1990−2021
	Number of cases	Age-standardized rate per 100000 population (95% UI)	Number of cases	Age-standardized rate per 100000 population (95% UI)	Estimated annual percentage changes (95% CI)
**Acute hepatitis A**					
**Global**	18869236	1380.3 (1378.3 to 1382.3)	19519113	1012.3 (1010.9 to 1013.7)	-1.11 (-1.17 to -1.05)
**Socio-demographic index**					
High	2081809	993.6 (989.4 to 997.8)	2264991	961.2 (957.2 to 965.2)	0.12 (0.02 to 0.22)
High-middle	3190176	1041.6 (1038 to 1045.2)	3306872	1181.8 (1177.7 to 1186)	0.08 (-0.03 to 0.19)
Middle	5411294	1129.1 (1126.1 to 1132.2)	6201146	1043.4 (1040.8 to 1046)	-0.3 (-0.36 to -0.25)
Low-middle	5969324	2284.9 (2278.9 to 2290.8)	5019486	972.3 (969.6 to 975)	-2.8 (-2.93 to -2.67)
Low	2201465	1973.1 (1964.5 to 1981.6)	2710005	928.8 (925.2 to 932.4)	-2.89 (-3.14 to -2.64)
**GBD regions**					
High-income Asia Pacific	385235	845 (837.2 to 852.9)	333813	907.8 (898.6 to 917)	0.44 (0.35 to 0.52)
Central Asia	199566	1086 (1071.9 to 1100.1)	253684	1080.3 (1067.9 to 1092.6)	0.16 (0.09 to 0.23)
East Asia	3950744	1098.4 (1095 to 1101.9)	3698725	1227.3 (1223.2 to 1231.4)	0.23 (0.18 to 0.27)
South Asia	6243106	2492.4 (2486 to 2498.7)	4402813	883.2 (880.6 to 885.8)	-3.53 (-3.75 to -3.3)
Southeast Asia	2041451	1641.4 (1634.2 to 1648.6)	2055599	1144.9 (1140 to 1149.9)	-1.21 (-1.33 to -1.1)
Australasia	47906	895.6 (875.5 to 915.8)	65423	928.8 (910.1 to 947.5)	0.22 (0.14 to 0.3)
Caribbean	105476	1040.5 (1023.7 to 1057.3)	142906	1198.4 (1181.4 to 1215.3)	0.56 (0.49 to 0.63)
Central Europe	358422	1206.3 (1195 to 1217.6)	276751	1169.2 (1156.7 to 1181.7)	-0.02 (-0.05 to 0.01)
Eastern Europe	639956	1186.7 (1178 to 1195.4)	517148	1211.4 (1201.4 to 1221.4)	0.14 (0.11 to 0.17)
Western Europe	866875	912.6 (906.9 to 918.3)	798686	877.6 (871.8 to 883.3)	-0.02 (-0.06 to 0.01)
Andean Latin America	78437	726.2 (712.9 to 739.6)	185425	1064.6 (1051 to 1078.3)	1.3 (1.24 to 1.35)
Central Latin America	344893	721.1 (714.2 to 728)	687787	1014.4 (1007.3 to 1021.6)	1.07 (0.98 to 1.16)
Southern Latin America	157006	1236.7 (1220.4 to 1253.1)	212612	1247 (1232.4 to 1261.6)	0.02 (-0.01 to 0.04)
Tropical Latin America	426076	977.6 (969 to 986.2)	708151	1217 (1208.4 to 1225.6)	0.71 (0.59 to 0.83)
North Africa and Middle East	959610	1194.3 (1186.5 to 1202.1)	1777167	1120.2 (1115 to 1125.4)	-0.32 (-0.4 to -0.23)
High-income North America	693980	947.6 (940.9 to 954.3)	805779	974.1 (967.7 to 980.6)	0.04 (-0.03 to 0.12)
Oceania	18978	1110.9 (1074.2 to 1147.6)	38940	1067.5 (1041 to 1094)	-0.12 (-0.13 to -0.1)
Central Sub-Saharan Africa	128874	950.1 (935 to 965.2)	363480	985.7 (975.9 to 995.5)	0.09 (-0.03 to 0.22)

Eastern Sub-Saharan Africa	591766	1447 (1434.9 to 1459.1)	843257	714.3 (709.3 to 719.2)	-2.78 (-3.02 to -2.53)
Southern Sub-Saharan Africa	69986	450.3 (440.3 to 460.3)	122781	558.5 (548.9 to 568)	0.61 (0.53 to 0.69)
Western Sub-Saharan Africa	560893	1356.4 (1344.6 to 1368.2)	1228186	897.9 (892.7 to 903)	-1.7 (-1.82 to -1.57)
**Acute hepatitis B**					
**Global**	17335420	1280.1 (1278.2 to 1282)	16787815	856.7 (855.4 to 858)	-1.24 (-1.33 to -1.15)
**Socio-demographic index**					
High	1261804	594.9 (591.7 to 598.2)	753371	290.3 (288.2 to 292.4)	-2.04 (-2.31 to -1.76)
High-middle	3791479	1256.8 (1252.8 to 1260.9)	2176489	657.9 (655.1 to 660.7)	-2.19 (-2.45 to -1.94)
Middle	7279527	1626.3 (1622.5 to 1630.1)	5551676	876.5 (874.2 to 878.8)	-1.87 (-1.96 to -1.79)
Low-middle	3036607	1135.8 (1131.7 to 1139.9)	4299681	855 (852.4 to 857.5)	-0.68 (-0.79 to -0.57)
Low	1955998	1655.6 (1648.2 to 1663)	3997145	1476.3 (1471.6 to 1480.9)	-0.46 (-0.52 to -0.4)
**GBD regions**					
High-income Asia Pacific	435977	959.1 (950.7 to 967.4)	221099	553.1 (546.5 to 559.7)	-1.98 (-2.07 to -1.88)
Central Asia	176505	1031.2 (1016.9 to 1045.4)	211914	839.4 (828.8 to 849.9)	-0.58 (-0.74 to -0.42)
East Asia	7744129	2296.2 (2291 to 2301.4)	3913092	1096.9 (1093.4 to 1100.5)	-2.28 (-2.43 to -2.13)
South Asia	2172508	849.3 (845.8 to 852.9)	3381138	683.3 (681 to 685.5)	-0.66 (-0.68 to -0.63)
Southeast Asia	1946850	1605.5 (1598.4 to 1612.7)	2035836	1097.4 (1092.7 to 1102.1)	-1.09 (-1.27 to -0.91)
Australasia	34376	640.2 (623.1 to 657.2)	30557	388.7 (377.7 to 399.8)	-1.45 (-1.67 to -1.22)
Caribbean	24604	258.3 (250.7 to 266)	24352	199.4 (193.5 to 205.3)	-0.7 (-0.79 to -0.61)
Central Europe	174028	577 (569.2 to 584.8)	70544	251.3 (246.2 to 256.3)	-2.46 (-2.67 to -2.25)
Eastern Europe	326277	584.8 (578.7 to 590.9)	188990	367.1 (362.1 to 372.1)	-1.07 (-1.48 to -0.67)
Western Europe	315094	329 (325.7 to 332.2)	187263	190.6 (188.2 to 193)	-1.64 (-1.8 to -1.48)
Andean Latin America	49401	526.7 (514.8 to 538.5)	75938	431.5 (423.4 to 439.6)	-0.55 (-0.62 to -0.47)
Central Latin America	227503	531.2 (524.8 to 537.6)	191651	276.8 (273.2 to 280.4)	-1.94 (-2.18 to -1.71)
Southern Latin America	19234	155.1 (150 to 160.3)	22806	129.7 (125.7 to 133.8)	-0.42 (-0.55 to -0.3)
Tropical Latin America	228455	570 (563.1 to 576.9)	154400	243.2 (239.7 to 246.7)	-2.67 (-2.97 to -2.37)
North Africa and Middle East	857781	1093 (1085.8 to 1100.2)	1064305	662.2 (658.4 to 666.1)	-1.58 (-1.73 to -1.44)
High-income North America	135774	181.9 (179.1 to 184.7)	91437	105.7 (103.7 to 107.7)	-1.51 (-1.79 to -1.22)
Oceania	35978	2309.8 (2251.4 to 2368.2)	55001	1598.8 (1564.5 to 1633)	-1.24 (-1.4 to -1.08)
Central Sub-Saharan Africa	326511	2622.9 (2596.3 to 2649.6)	782151	2410.5 (2394 to 2426.9)	-0.34 (-0.41 to -0.27)
Eastern Sub-Saharan Africa	707023	1637.9 (1626.1 to 1649.7)	1267775	1223.8 (1217.1 to 1230.4)	-0.83 (-0.93 to -0.73)
Southern Sub-Saharan Africa	207021	1514.9 (1495.7 to 1534.1)	214720	977.5 (965.4 to 989.6)	-1.4 (-1.52 to -1.28)
Western Sub-Saharan Africa	1190391	2732.3 (2717 to 2747.7)	2602847	2225.1 (2216.5 to 2233.7)	-0.61 (-0.67 to -0.54)

**Acute hepatitis C**					
**Global**	652543	50.3 (50 to 50.7)	931188	47.5 (47.2 to 47.8)	-0.18 (-0.29 to -0.07)
**Socio-demographic index**					
High	91818	42.6 (41.9 to 43.4)	91639	35.6 (35 to 36.3)	-0.49 (-0.64 to -0.34)
High-middle	102914	35.1 (34.4 to 35.7)	95767	30.4 (29.8 to 31)	-0.99 (-1.25 to -0.74)
Middle	193158	44.6 (44 to 45.1)	250746	40.1 (39.6 to 40.5)	-0.31 (-0.45 to -0.16)
Low-middle	175164	70 (69 to 71.1)	296920	60.2 (59.5 to 60.8)	-0.22 (-0.4 to -0.04)
Low	88938	81.6 (80.2 to 83.1)	195347	76.4 (75.5 to 77.4)	-0.36 (-0.41 to -0.31)
**GBD regions**					
High-income Asia Pacific	33204	69.8 (67.6 to 72)	16647	37.7 (36.1 to 39.3)	-3.21 (-4.05 to -2.37)
Central Asia	11073	65.8 (63.1 to 68.5)	17828	73.6 (71.1 to 76.1)	0.37 (0.31 to 0.43)
East Asia	96230	28.7 (28.2 to 29.3)	57365	16.9 (16.5 to 17.3)	-2.49 (-2.96 to -2.02)
South Asia	76266	31.1 (30.5 to 31.8)	132116	27.2 (26.7 to 27.6)	-0.22 (-0.52 to 0.09)
Southeast Asia	61634	51.3 (50.3 to 52.3)	90102	49 (48.1 to 49.8)	-0.1 (-0.14 to -0.06)
Australasia	2260	40.8 (37.5 to 44.2)	3264	43.6 (40.5 to 46.7)	0.43 (0.34 to 0.52)
Caribbean	5451	61.5 (58.1 to 64.8)	6756	55.9 (53.1 to 58.7)	-0.33 (-0.4 to -0.27)
Central Europe	7960	26.2 (25 to 27.4)	5850	21.8 (20.6 to 22.9)	-0.52 (-0.56 to -0.49)
Eastern Europe	20922	37.9 (36.7 to 39.2)	22815	47.6 (46 to 49.1)	0.86 (0.67 to 1.05)
Western Europe	38253	39.2 (38.2 to 40.2)	30102	30 (29.2 to 30.9)	-0.89 (-0.95 to -0.84)
Andean Latin America	3425	38.9 (36.2 to 41.5)	5576	32.5 (30.7 to 34.3)	-0.49 (-0.59 to -0.39)
Central Latin America	25717	62.3 (60.3 to 64.3)	38444	56.5 (55 to 58)	0.14 (-0.1 to 0.38)
Southern Latin America	3599	28.9 (26.9 to 30.9)	4457	25.2 (23.6 to 26.8)	-0.16 (-0.34 to 0.01)
Tropical Latin America	32193	83.7 (81 to 86.3)	46715	75.3 (73.3 to 77.3)	0.34 (-0.06 to 0.74)
North Africa and Middle East	105417	151.3 (148.5 to 154.1)	190641	119.6 (118 to 121.3)	-0.67 (-0.83 to -0.51)
High-income North America	22309	29.3 (28.3 to 30.3)	27433	31.9 (30.9 to 32.9)	0.37 (0.2 to 0.55)
Oceania	470	31.2 (26.7 to 35.8)	1112	32.4 (29 to 35.8)	0.13 (0.11 to 0.16)
Central Sub-Saharan Africa	17530	160.7 (155.3 to 166.1)	41883	142 (138.6 to 145.4)	-0.52 (-0.57 to -0.47)
Eastern Sub-Saharan Africa	40853	107.9 (105.3 to 110.5)	84465	87.7 (86.1 to 89.2)	-0.77 (-0.82 to -0.72)
Southern Sub-Saharan Africa	7314	57.4 (54.6 to 60.3)	12934	60.2 (57.8 to 62.5)	0.12 (0 to 0.24)
Western Sub-Saharan Africa	40461	96.1 (93.8 to 98.4)	94681	82 (80.5 to 83.4)	-0.61 (-0.69 to -0.54)
**Acute hepatitis E**					
**Global**	2727119	190.6 (189.9 to 191.3)	3420786	178.2 (177.6 to 178.8)	-0.34 (-0.38 to -0.29)

**Socio-demographic index**					
High	190820	91.9 (90.7 to 93)	192231	82.5 (81.5 to 83.6)	-0.11 (-0.25 to 0.02)
High-middle	566674	184.6 (183.1 to 186.1)	428671	154.7 (153.2 to 156.2)	-0.88 (-1.05 to -0.72)
Middle	1056945	216.5 (215.2 to 217.7)	1072583	180.4 (179.4 to 181.5)	-0.68 (-0.74 to -0.63)
Low-middle	661350	221.9 (220.3 to 223.5)	1129814	214 (212.8 to 215.2)	-0.21 (-0.3 to -0.11)
Low	249252	190.3 (188.1 to 192.4)	595157	194.8 (193.3 to 196.3)	-0.15 (-0.21 to -0.08)
**GBD regions**					
High-income Asia Pacific	42033	93 (90.8 to 95.2)	31397	90.5 (88 to 92.9)	-0.05 (-0.1 to 0)
Central Asia	26501	149.2 (144.9 to 153.4)	34814	145.9 (142.2 to 149.6)	-0.12 (-0.14 to -0.1)
East Asia	1091527	298.9 (297.1 to 300.6)	665367	230 (228.2 to 231.8)	-1.04 (-1.17 to -0.91)
South Asia	783812	276.6 (274.7 to 278.4)	1403294	274.4 (273 to 275.8)	-0.34 (-0.46 to -0.22)
Southeast Asia	175138	138 (136.2 to 139.7)	245256	135.4 (133.9 to 136.8)	0.02 (-0.01 to 0.04)
Australasia	3175	59.6 (55.6 to 63.6)	4180	60.5 (56.8 to 64.2)	0.04 (0.04 to 0.05)
Caribbean	21392	213.6 (206.9 to 220.4)	24831	209 (202.8 to 215.2)	-0.14 (-0.22 to -0.07)
Central Europe	49070	165.6 (162 to 169.3)	36563	157.2 (153.2 to 161.1)	-0.13 (-0.15 to -0.12)
Eastern Europe	44040	80.6 (78.8 to 82.5)	36846	79.5 (77.5 to 81.5)	0 (-0.03 to 0.02)
Western Europe	44069	46.4 (45.3 to 47.5)	41949	45.9 (44.8 to 47)	0.03 (-0.05 to 0.11)
Andean Latin America	16234	159 (153.5 to 164.5)	26504	151.8 (147.4 to 156.1)	-0.15 (-0.17 to -0.12)
Central Latin America	44228	101.3 (98.9 to 103.6)	68765	101 (99.1 to 102.9)	0.06 (0.03 to 0.09)
Southern Latin America	4769	38 (35.9 to 40.2)	6202	35.8 (34 to 37.7)	-0.14 (-0.16 to -0.12)
Tropical Latin America	27927	68.2 (66.3 to 70.1)	40900	68.6 (66.9 to 70.2)	0.09 (0.04 to 0.13)
North Africa and Middle East	86424	103.6 (101.8 to 105.4)	156314	98.1 (96.7 to 99.4)	-0.22 (-0.26 to -0.18)
High-income North America	46831	64.4 (63 to 65.8)	54861	66.5 (65.1 to 67.9)	0.12 (0.11 to 0.14)
Oceania	2613	156.8 (145.3 to 168.2)	5482	152.4 (143.9 to 160.9)	-0.1 (-0.11 to -0.08)
Central Sub-Saharan Africa	29165	206 (200.4 to 211.6)	75383	204 (200.2 to 207.8)	-0.12 (-0.15 to -0.09)
Eastern Sub-Saharan Africa	87478	181.7 (178.5 to 184.8)	210682	177.2 (175.1 to 179.3)	-0.08 (-0.09 to -0.06)
Southern Sub-Saharan Africa	20794	141.5 (137 to 146.1)	33488	152.9 (148.8 to 156.9)	0.35 (0.29 to 0.41)
Western Sub-Saharan Africa	79899	165 (162.1 to 167.9)	217710	164.3 (162.4 to 166.3)	-0.04 (-0.05 to -0.03)
**Chronic hepatitis B**					
**Global**	1109657	80.6 (80.2 to 81.1)	1020188	52.2 (51.9 to 52.5)	-1.33 (-1.44 to -1.22)
**Socio-demographic index**					
High	78566	37.4 (36.7 to 38.1)	44373	17.5 (17.1 to 17.9)	-2.26 (-2.54 to -1.98)
High-middle	239801	79.2 (78.2 to 80.2)	125715	38.2 (37.6 to 38.8)	-2.4 (-2.71 to -2.1)

Middle	468093	102 (101.1 to 102.9)	330632	52.3 (51.8 to 52.8)	-2.01 (-2.12 to -1.89)
Low-middle	195603	71.1 (70.2 to 72)	263970	52.1 (51.5 to 52.7)	-0.74 (-0.87 to -0.61)
Low	126955	103.9 (102.3 to 105.5)	254929	91.5 (90.4 to 92.5)	-0.49 (-0.56 to -0.43)
**GBD regions**					
High-income Asia Pacific	27291	60.2 (58.4 to 61.9)	13313	33.8 (32.5 to 35.2)	-2.04 (-2.12 to -1.96)
Central Asia	11261	65.2 (62.5 to 68)	12442	48.3 (46.4 to 50.3)	-0.74 (-0.94 to -0.54)
East Asia	494251	143.9 (142.6 to 145.2)	226587	64 (63.2 to 64.8)	-2.47 (-2.67 to -2.26)
South Asia	139298	53.2 (52.5 to 54)	211250	42.4 (41.9 to 42.9)	-0.67 (-0.7 to -0.64)
Southeast Asia	125168	100.6 (99.1 to 102.2)	121745	65.6 (64.6 to 66.6)	-1.24 (-1.45 to -1.04)
Australasia	2152	40.3 (37.1 to 43.6)	1764	22.1 (20.1 to 24)	-1.71 (-1.98 to -1.44)
Caribbean	1586	16 (14.6 to 17.5)	1491	12.1 (11 to 13.3)	-0.82 (-0.92 to -0.72)
Central Europe	10847	36.3 (34.8 to 37.9)	4079	14.9 (14 to 15.8)	-2.7 (-2.94 to -2.46)
Eastern Europe	20205	36.4 (35.1 to 37.7)	10914	21.6 (20.6 to 22.5)	-1.24 (-1.7 to -0.79)
Western Europe	19646	20.6 (20 to 21.3)	11129	11.5 (11 to 11.9)	-1.82 (-1.98 to -1.66)
Andean Latin America	3167	32.8 (30.6 to 35.1)	4541	25.8 (24.2 to 27.3)	-0.63 (-0.74 to -0.53)
Central Latin America	14810	33.2 (31.9 to 34.4)	11308	16.1 (15.4 to 16.7)	-2.16 (-2.43 to -1.89)
Southern Latin America	1210	9.7 (8.7 to 10.6)	1336	7.8 (7 to 8.6)	-0.55 (-0.7 to -0.39)
Tropical Latin America	14595	35.7 (34.3 to 37.1)	8895	13.7 (13 to 14.4)	-2.91 (-3.27 to -2.55)
North Africa and Middle East	55217	68 (66.6 to 69.5)	63019	38.9 (38.1 to 39.7)	-1.75 (-1.93 to -1.58)
High-income North America	8366	11.4 (10.9 to 12)	5404	6.4 (6.1 to 6.8)	-1.75 (-2.05 to -1.45)
Oceania	2324	145 (133.8 to 156.2)	3316	94.3 (87.9 to 100.7)	-1.41 (-1.59 to -1.24)
Central Sub-Saharan Africa	21194	163.6 (158.4 to 168.8)	50521	150.1 (146.7 to 153.5)	-0.34 (-0.4 to -0.28)
Eastern Sub-Saharan Africa	46156	102.8 (100.4 to 105.2)	79310	74.6 (73.2 to 76)	-0.89 (-1.02 to -0.76)
Southern Sub-Saharan Africa	13492	96.6 (92.9 to 100.3)	12966	58.5 (56.2 to 60.8)	-1.55 (-1.69 to -1.4)
Western Sub-Saharan Africa	77421	170.7 (167.6 to 173.8)	164858	136.3 (134.5 to 138.1)	-0.64 (-0.72 to -0.57)
**Chronic hepatitis C**					
**Global**	431285	33.3 (33 to 33.6)	587617	30 (29.8 to 30.2)	-0.29 (-0.38 to -0.2)
**Socio-demographic index**					
High	60675	28.2 (27.5 to 28.8)	58439	22.8 (22.3 to 23.3)	-0.59 (-0.73 to -0.46)
High-middle	68004	23.2 (22.7 to 23.7)	62113	19.7 (19.2 to 20.2)	-1.03 (-1.27 to -0.78)
Middle	127642	29.5 (29 to 29.9)	164054	26.2 (25.8 to 26.6)	-0.33 (-0.47 to -0.18)
Low-middle	115815	46.3 (45.5 to 47.1)	174536	35.1 (34.6 to 35.6)	-0.53 (-0.77 to -0.28)
Low	58784	53.9 (52.8 to 55.1)	127988	50 (49.3 to 50.8)	-0.37 (-0.42 to -0.33)

**GBD regions**					
High-income Asia Pacific	21945	46.1 (44.4 to 47.9)	10216	23.2 (22 to 24.5)	-3.54 (-4.4 to -2.67)
Central Asia	7317	43.4 (41.3 to 45.5)	11526	47.7 (45.8 to 49.6)	0.33 (0.26 to 0.4)
East Asia	63586	19 (18.6 to 19.5)	36766	10.8 (10.5 to 11.1)	-2.54 (-2.98 to -2.09)
South Asia	50396	20.6 (20 to 21.1)	83531	17.2 (16.8 to 17.5)	-0.29 (-0.61 to 0.03)
Southeast Asia	40731	33.9 (33.1 to 34.7)	59514	32.3 (31.7 to 33)	-0.1 (-0.14 to -0.06)
Australasia	1493	27 (24.4 to 29.7)	1724	23.3 (21.1 to 25.5)	-0.19 (-0.35 to -0.02)
Caribbean	3602	40.7 (38 to 43.3)	4465	36.9 (34.7 to 39.1)	-0.34 (-0.41 to -0.27)
Central Europe	5260	17.3 (16.3 to 18.3)	3669	13.8 (12.9 to 14.7)	-0.6 (-0.65 to -0.55)
Eastern Europe	13826	25 (24 to 26)	14821	30.9 (29.7 to 32.1)	0.83 (0.64 to 1.03)
Western Europe	25277	25.9 (25.1 to 26.7)	19310	19.2 (18.6 to 19.9)	-0.96 (-1.02 to -0.89)
Andean Latin America	2263	25.6 (23.6 to 27.6)	3684	21.5 (20.1 to 22.9)	-0.49 (-0.59 to -0.4)
Central Latin America	16995	41 (39.5 to 42.6)	25269	37.2 (36 to 38.3)	0.13 (-0.1 to 0.36)
Southern Latin America	2378	19 (17.5 to 20.6)	2898	16.4 (15.2 to 17.7)	-0.19 (-0.36 to -0.02)
Tropical Latin America	21275	55.3 (53.2 to 57.4)	29923	48.1 (46.5 to 49.6)	0.28 (-0.13 to 0.7)
North Africa and Middle East	69723	100 (97.8 to 102.1)	107472	67.3 (66.1 to 68.5)	-1.09 (-1.24 to -0.95)
High-income North America	14741	19.2 (18.5 to 20)	17832	20.7 (20 to 21.5)	0.35 (0.16 to 0.53)
Oceania	311	20.3 (16.8 to 23.8)	735	21.2 (18.6 to 23.9)	0.16 (0.14 to 0.18)
Central Sub-Saharan Africa	11590	106.4 (102.1 to 110.6)	27687	93.7 (91 to 96.4)	-0.52 (-0.57 to -0.47)
Eastern Sub-Saharan Africa	27002	71.4 (69.3 to 73.4)	55463	57.6 (56.4 to 58.8)	-0.78 (-0.83 to -0.74)
Southern Sub-Saharan Africa	4834	37.9 (35.7 to 40.1)	8542	39.7 (37.9 to 41.6)	0.12 (0 to 0.23)
Western Sub-Saharan Africa	26741	63.4 (61.6 to 65.3)	62571	54.1 (53 to 55.2)	-0.61 (-0.68 to -0.54)

Supplementary Table 3. Global prevalence of hepatitis in women aged 15–49 years in 1990 and 2021, and estimated annual percentage changes from 1990 to 2021

Characteristics	1990		2021		1990−2021
	Number of cases	Age-standardized rate per 100000 population (95% UI)	Number of cases	Age-standardized rate per 100000 population (95% UI)	Estimated annual percentage changes (95% CI)
**Acute hepatitis A**					
**Global**	898481	63.1 (62.7 to 63.5)	1399533	72.8 (72.4 to 73.2)	0.46 (0.42 to 0.49)
**Socio-demographic index**					
High	159976	76.4 (75.3 to 77.4)	174230	73.9 (72.9 to 74.9)	0.12 (0.03 to 0.22)
High-middle	239323	78 (77 to 78.9)	254071	91.1 (90 to 92.2)	0.16 (0.06 to 0.26)
Middle	330783	66.5 (65.9 to 67.2)	472859	79.7 (79 to 80.4)	0.56 (0.52 to 0.6)
Low-middle	117125	38.4 (37.8 to 39)	323390	61.3 (60.6 to 61.9)	1.79 (1.66 to 1.91)
Low	50219	36.9 (36 to 37.7)	173728	55.4 (54.7 to 56.2)	1.16 (1.1 to 1.22)
**GBD regions**					
High-income Asia Pacific	29633	64.7 (62.9 to 66.5)	25678	69.8 (67.7 to 71.9)	0.44 (0.36 to 0.51)
Central Asia	14022	75.1 (72.3 to 77.9)	19514	82.8 (80.1 to 85.6)	0.33 (0.31 to 0.35)
East Asia	291768	79.9 (79 to 80.7)	284517	94.4 (93.4 to 95.5)	0.33 (0.28 to 0.39)
South Asia	58458	20.2 (19.7 to 20.6)	260311	51.1 (50.5 to 51.6)	3.54 (3.31 to 3.77)
Southeast Asia	103941	77.2 (75.9 to 78.4)	152599	85.4 (84.2 to 86.5)	0.32 (0.29 to 0.34)
Australasia	3685	68.7 (64.3 to 73)	5033	71.7 (67.6 to 75.9)	0.23 (0.16 to 0.3)
Caribbean	8073	78.7 (74.9 to 82.4)	10993	92 (88.1 to 95.9)	0.57 (0.51 to 0.64)
Central Europe	27571	92.6 (90 to 95.2)	21289	90.3 (87.5 to 93.2)	-0.02 (-0.04 to 0.01)
Eastern Europe	49227	91.2 (89.2 to 93.3)	39781	93.1 (90.8 to 95.4)	0.14 (0.11 to 0.17)
Western Europe	66683	70 (68.7 to 71.4)	61437	67.6 (66.2 to 68.9)	-0.02 (-0.06 to 0.01)
Andean Latin America	6034	55.4 (52.5 to 58.3)	14263	82.4 (79.3 to 85.6)	1.29 (1.24 to 1.35)
Central Latin America	26525	55.5 (53.9 to 57.1)	52907	78.2 (76.5 to 79.9)	1.07 (0.98 to 1.16)
Southern Latin America	12077	95 (91.3 to 98.7)	16355	95.8 (92.5 to 99.2)	0.02 (-0.01 to 0.04)
Tropical Latin America	32770	74.9 (72.9 to 76.8)	54473	93.4 (91.4 to 95.4)	0.71 (0.59 to 0.82)
North Africa and Middle East	51681	56.7 (55.4 to 57.9)	127643	80.3 (79.1 to 81.5)	1.06 (0.94 to 1.18)
High-income North America	53383	73.4 (71.8 to 75)	61983	74.9 (73.4 to 76.4)	0.04 (-0.02 to 0.11)
Oceania	1434	82.2 (74.5 to 89.9)	2993	81.7 (75.8 to 87.6)	-0.03 (-0.05 to -0.01)
Central Sub-Saharan Africa	8975	62.2 (59.5 to 65)	27893	75.4 (73.2 to 77.6)	0.56 (0.49 to 0.63)

Eastern Sub-Saharan Africa	17854	35.2 (33.9 to 36.5)	58201	46.6 (45.6 to 47.6)	0.54 (0.4 to 0.68)
Southern Sub-Saharan Africa	5265	34.2 (32.3 to 36.1)	9346	42.5 (40.6 to 44.4)	0.6 (0.56 to 0.63)
Western Sub-Saharan Africa	29421	57.2 (55.5 to 58.9)	92326	66.8 (65.6 to 68.1)	0.43 (0.32 to 0.55)
**Acute hepatitis B**					
**Global**	2000241	147.7 (147.1 to 148.4)	1937056	98.8 (98.4 to 99.3)	-1.24 (-1.33 to -1.15)
**Socio-demographic index**					
High	145593	68.6 (67.6 to 69.6)	86927	33.7 (33 to 34.3)	-2.04 (-2.31 to -1.76)
High-middle	437478	145 (143.7 to 146.4)	251133	75.8 (74.9 to 76.7)	-2.19 (-2.45 to -1.94)
Middle	839945	187.7 (186.4 to 188.9)	640578	100.9 (100.2 to 101.7)	-1.87 (-1.96 to -1.79)
Low-middle	350378	131.1 (129.8 to 132.4)	496117	98.7 (97.9 to 99.5)	-0.68 (-0.79 to -0.57)
Low	225692	191 (188.7 to 193.3)	461209	170.6 (169.1 to 172)	-0.46 (-0.51 to -0.4)
**GBD regions**					
High-income Asia Pacific	50305	110.7 (108.3 to 113.2)	25511	63.3 (61.5 to 65.2)	-1.98 (-2.07 to -1.89)
Central Asia	20366	119.8 (115.7 to 123.8)	24452	95.3 (92.3 to 98.3)	-0.59 (-0.74 to -0.43)
East Asia	893553	264.9 (263.2 to 266.7)	451511	126.3 (125.2 to 127.5)	-2.28 (-2.43 to -2.13)
South Asia	250674	98.1 (97 to 99.2)	390131	78.9 (78.2 to 79.6)	-0.66 (-0.68 to -0.63)
Southeast Asia	224637	185.1 (182.9 to 187.3)	234904	126.5 (125.1 to 128)	-1.09 (-1.27 to -0.91)
Australasia	3966	74.1 (69.5 to 78.7)	3526	44.3 (41.3 to 47.2)	-1.44 (-1.67 to -1.22)
Caribbean	2839	29.5 (27.4 to 31.6)	2810	22.9 (21.2 to 24.5)	-0.71 (-0.79 to -0.62)
Central Europe	20080	66.8 (64.6 to 69)	8140	29.5 (28.2 to 30.8)	-2.46 (-2.66 to -2.25)
Eastern Europe	37647	67.1 (65.3 to 68.9)	21807	42.7 (41.2 to 44.1)	-1.08 (-1.48 to -0.67)
Western Europe	36357	37.9 (36.9 to 38.8)	21607	22 (21.3 to 22.7)	-1.64 (-1.81 to -1.48)
Andean Latin America	5700	61 (57.7 to 64.3)	8762	49.9 (47.6 to 52.2)	-0.55 (-0.62 to -0.48)
Central Latin America	26250	60.9 (59.1 to 62.7)	22114	31.5 (30.5 to 32.5)	-1.94 (-2.17 to -1.71)
Southern Latin America	2219	17.9 (16.5 to 19.3)	2631	15.2 (14 to 16.3)	-0.42 (-0.54 to -0.3)
Tropical Latin America	26360	65.8 (63.8 to 67.8)	17815	27.7 (26.7 to 28.7)	-2.67 (-2.97 to -2.38)
North Africa and Middle East	98975	126.1 (124 to 128.3)	122804	76.1 (74.9 to 77.3)	-1.58 (-1.73 to -1.43)
High-income North America	15666	21.1 (20.3 to 21.8)	10550	12.4 (11.9 to 13)	-1.51 (-1.78 to -1.23)
Oceania	4151	267.7 (251.4 to 284)	6346	182.9 (173.3 to 192.4)	-1.24 (-1.4 to -1.09)
Central Sub-Saharan Africa	37674	301.8 (294.3 to 309.3)	90248	277.8 (273 to 282.7)	-0.34 (-0.4 to -0.28)
Eastern Sub-Saharan Africa	81580	189.3 (185.8 to 192.7)	146282	141.7 (139.7 to 143.7)	-0.83 (-0.92 to -0.73)
Southern Sub-Saharan Africa	23887	176.6 (171.2 to 182.1)	24775	112.1 (108.8 to 115.5)	-1.4 (-1.52 to -1.28)
Western Sub-Saharan Africa	137353	315 (310.4 to 319.6)	300329	256.6 (253.9 to 259.2)	-0.61 (-0.67 to -0.54)

**Acute hepatitis C**					
**Global**	75293	5.8 (5.7 to 5.9)	107445	5.5 (5.4 to 5.6)	-0.18 (-0.28 to -0.08)
**Socio-demographic index**					
High	10594	4.9 (4.7 to 5.1)	10574	4.2 (4 to 4.3)	-0.49 (-0.63 to -0.35)
High-middle	11875	4 (3.9 to 4.2)	11050	3.4 (3.3 to 3.6)	-1 (-1.24 to -0.76)
Middle	22287	5.2 (5 to 5.3)	28932	4.6 (4.5 to 4.7)	-0.31 (-0.45 to -0.17)
Low-middle	20211	8.1 (7.8 to 8.4)	34260	7 (6.8 to 7.2)	-0.22 (-0.39 to -0.05)
Low	10262	9.4 (9 to 9.8)	22540	8.7 (8.4 to 9)	-0.36 (-0.4 to -0.32)
**GBD regions**					
High-income Asia Pacific	3831	8.1 (7.5 to 8.8)	1921	4.4 (3.9 to 4.8)	-3.23 (-4.04 to -2.41)
Central Asia	1278	7.5 (6.8 to 8.3)	2057	8.5 (7.8 to 9.2)	0.39 (0.33 to 0.45)
East Asia	11103	3.4 (3.2 to 3.5)	6619	1.9 (1.8 to 2)	-2.52 (-2.97 to -2.07)
South Asia	8800	3.6 (3.4 to 3.8)	15244	3.1 (3 to 3.3)	-0.22 (-0.51 to 0.07)
Southeast Asia	7112	5.9 (5.6 to 6.2)	10396	5.6 (5.4 to 5.9)	-0.1 (-0.14 to -0.06)
Australasia	261	4.6 (3.8 to 5.5)	377	5.1 (4.3 to 5.9)	0.37 (0.31 to 0.43)
Caribbean	629	7.1 (6.1 to 8)	780	6.4 (5.7 to 7.2)	-0.32 (-0.38 to -0.26)
Central Europe	918	3 (2.7 to 3.4)	675	2.6 (2.2 to 2.9)	-0.52 (-0.55 to -0.49)
Eastern Europe	2414	4.3 (4 to 4.7)	2632	5.5 (5 to 5.9)	0.87 (0.7 to 1.04)
Western Europe	4414	4.5 (4.2 to 4.8)	3473	3.4 (3.2 to 3.7)	-0.89 (-0.93 to -0.85)
Andean Latin America	395	4.4 (3.7 to 5.1)	643	3.8 (3.3 to 4.3)	-0.47 (-0.52 to -0.41)
Central Latin America	2967	7 (6.5 to 7.6)	4436	6.5 (6.1 to 7)	0.14 (-0.07 to 0.35)
Southern Latin America	415	3.2 (2.7 to 3.7)	514	3 (2.5 to 3.4)	-0.19 (-0.31 to -0.08)
Tropical Latin America	3715	9.7 (9 to 10.5)	5390	8.6 (8 to 9.2)	0.34 (-0.03 to 0.71)
North Africa and Middle East	12164	17.3 (16.6 to 18.1)	21997	13.9 (13.5 to 14.4)	-0.67 (-0.81 to -0.53)
High-income North America	2574	3.3 (3 to 3.5)	3165	3.6 (3.4 to 3.9)	0.36 (0.21 to 0.52)
Oceania	54	4.5 (2.9 to 6)	128	3.9 (2.9 to 4.9)	-0.42 (-0.62 to -0.22)
Central Sub-Saharan Africa	2023	18.6 (17.1 to 20.2)	4833	16.2 (15.3 to 17.2)	-0.52 (-0.56 to -0.48)
Eastern Sub-Saharan Africa	4714	12.5 (11.8 to 13.2)	9746	10.1 (9.6 to 10.5)	-0.77 (-0.82 to -0.73)
Southern Sub-Saharan Africa	844	6.6 (5.8 to 7.4)	1492	6.9 (6.2 to 7.5)	0.09 (-0.02 to 0.2)
Western Sub-Saharan Africa	4669	11 (10.4 to 11.7)	10925	9.4 (9 to 9.8)	-0.61 (-0.68 to -0.54)
**Acute hepatitis E**					
**Global**	209778	14.7 (14.5 to 14.9)	263137	13.7 (13.5 to 13.8)	-0.34 (-0.38 to -0.29)

**Socio-demographic index**					
High	14678	7.1 (6.8 to 7.3)	14787	6.4 (6.2 to 6.7)	-0.11 (-0.24 to 0.02)
High-middle	43590	14.2 (13.8 to 14.6)	32975	11.8 (11.5 to 12.2)	-0.88 (-1.03 to -0.73)
Middle	81303	16.7 (16.4 to 17)	82506	13.8 (13.6 to 14.1)	-0.68 (-0.73 to -0.63)
Low-middle	50873	17.1 (16.7 to 17.5)	86909	16.5 (16.2 to 16.8)	-0.2 (-0.3 to -0.11)
Low	19173	14.7 (14.2 to 15.2)	45781	14.9 (14.5 to 15.2)	-0.15 (-0.2 to -0.09)
**GBD regions**					
High-income Asia Pacific	3233	7.2 (6.7 to 7.7)	2415	7 (6.4 to 7.5)	-0.05 (-0.09 to -0.01)
Central Asia	2039	11.5 (10.5 to 12.4)	2678	11.2 (10.4 to 12)	-0.12 (-0.14 to -0.11)
East Asia	83964	23 (22.5 to 23.4)	51182	17.7 (17.2 to 18.1)	-1.04 (-1.16 to -0.92)
South Asia	60293	21.4 (20.9 to 21.8)	107946	21.1 (20.7 to 21.4)	-0.34 (-0.46 to -0.23)
Southeast Asia	13472	10.6 (10.2 to 11)	18866	10.4 (10.1 to 10.8)	0.02 (-0.01 to 0.04)
Australasia	244	4.6 (3.7 to 5.4)	322	4.9 (4 to 5.8)	0.22 (0.22 to 0.23)
Caribbean	1646	16.4 (14.9 to 17.9)	1910	16 (14.6 to 17.3)	-0.15 (-0.21 to -0.09)
Central Europe	3775	12.8 (12 to 13.6)	2813	12.2 (11.3 to 13.1)	-0.13 (-0.14 to -0.12)
Eastern Europe	3388	6.2 (5.8 to 6.6)	2834	6.1 (5.7 to 6.6)	0 (-0.01 to 0.02)
Western Europe	3390	3.6 (3.3 to 3.8)	3227	3.5 (3.3 to 3.8)	0.03 (-0.04 to 0.11)
Andean Latin America	1249	12.3 (11.1 to 13.6)	2039	11.7 (10.7 to 12.7)	-0.16 (-0.19 to -0.14)
Central Latin America	3402	7.7 (7.2 to 8.2)	5290	7.8 (7.3 to 8.2)	0.07 (0.05 to 0.09)
Southern Latin America	367	2.9 (2.4 to 3.4)	477	2.7 (2.3 to 3.1)	-0.16 (-0.18 to -0.14)
Tropical Latin America	2148	5.2 (4.8 to 5.6)	3146	5.3 (4.9 to 5.7)	0.1 (0.06 to 0.13)
North Africa and Middle East	6648	7.9 (7.5 to 8.3)	12024	7.5 (7.2 to 7.8)	-0.22 (-0.25 to -0.19)
High-income North America	3602	4.9 (4.6 to 5.3)	4220	5.1 (4.8 to 5.4)	0.13 (0.12 to 0.14)
Oceania	201	12.3 (9.7 to 14.8)	422	11.9 (10 to 13.8)	-0.12 (-0.16 to -0.08)
Central Sub-Saharan Africa	2243	15.9 (14.7 to 17.2)	5799	15.6 (14.7 to 16.5)	-0.12 (-0.15 to -0.1)
Eastern Sub-Saharan Africa	6729	14 (13.3 to 14.7)	16206	13.6 (13.1 to 14.1)	-0.08 (-0.09 to -0.07)
Southern Sub-Saharan Africa	1600	10.8 (9.8 to 11.8)	2576	11.8 (10.9 to 12.7)	0.35 (0.31 to 0.39)
Western Sub-Saharan Africa	6146	12.7 (12.1 to 13.4)	16747	12.6 (12.2 to 13.1)	-0.04 (-0.05 to -0.03)
**Chronic hepatitis B**					
**Global**	72138954	5350.1 (5337.7 to 5362.4)	71396795	3642.6 (3634.2 to 3651)	-1.22 (-1.3 to -1.13)
**Socio-demographic index**					
High	4087918	1926.6 (1908.6 to 1944.6)	2692639	1021.1 (1009.4 to 1032.9)	-1.77 (-2.02 to -1.52)
High-middle	15644745	5221.8 (5195.9 to 5247.7)	10282850	3059.2 (3040.3 to 3078.1)	-1.9 (-2.08 to -1.72)

Middle	30636313	6901.3 (6876.6 to 6926)	23432534	3686.5 (3671.7 to 3701.2)	-2.03 (-2.1 to -1.95)
Low-middle	12566466	4694.7 (4669.2 to 4720.2)	17187600	3424.2 (3408.3 to 3440.1)	-0.73 (-0.86 to -0.6)
Low	9163269	7735 (7685.6 to 7784.4)	17762647	6543.6 (6513.1 to 6574.1)	-0.6 (-0.68 to -0.52)
**GBD regions**					
High-income Asia Pacific	1277748	2797.8 (2755.9 to 2839.7)	644440	1519.5 (1488.5 to 1550.4)	-2.12 (-2.22 to -2.03)
Central Asia	643965	3797.8 (3717.2 to 3878.3)	788844	3129.9 (3069.8 to 3189.9)	-0.52 (-0.69 to -0.35)
East Asia	32969323	9882.9 (9848.5 to 9917.3)	18888528	5188.8 (5164.9 to 5212.8)	-2.15 (-2.26 to -2.05)
South Asia	9191314	3588.2 (3566.3 to 3610.1)	14107542	2857 (2842.8 to 2871.2)	-0.62 (-0.66 to -0.58)
Southeast Asia	7747648	6430.9 (6387.4 to 6474.4)	7690107	4140.2 (4112.6 to 4167.9)	-1.34 (-1.49 to -1.2)
Australasia	119685	2227.3 (2136.6 to 2318.1)	102699	1299.3 (1242.2 to 1356.4)	-1.56 (-1.77 to -1.34)
Caribbean	105355	1119.3 (1071.5 to 1167.1)	103502	850.2 (813.7 to 886.7)	-0.76 (-0.85 to -0.67)
Central Europe	580825	1921.2 (1880.3 to 1962)	260857	905.7 (879 to 932.3)	-2.25 (-2.49 to -2.01)
Eastern Europe	1428924	2576.3 (2537 to 2615.5)	857840	1625.4 (1594 to 1656.8)	-1.22 (-1.53 to -0.91)
Western Europe	1024795	1067.8 (1050.5 to 1085.2)	628393	622.3 (609.9 to 634.7)	-1.62 (-1.77 to -1.47)
Andean Latin America	125025	1349.2 (1296.3 to 1402.1)	180915	1025.6 (990.8 to 1060.4)	-0.82 (-0.88 to -0.76)
Central Latin America	1158983	2751.1 (2708.3 to 2793.9)	978989	1414.1 (1390.6 to 1437.6)	-2.02 (-2.24 to -1.81)
Southern Latin America	49504	399.4 (376.7 to 422.2)	55405	311 (293.9 to 328.1)	-0.59 (-0.71 to -0.47)
Tropical Latin America	799577	2007.1 (1969.6 to 2044.5)	695323	1078.7 (1057.4 to 1100.1)	-1.86 (-2.14 to -1.58)
North Africa and Middle East	3109221	3940.5 (3900.2 to 3980.9)	3482294	2168.7 (2147.9 to 2189.5)	-1.83 (-1.98 to -1.67)
High-income North America	448438	602.3 (588.2 to 616.3)	329287	382.9 (372.8 to 393)	-1.32 (-1.54 to -1.11)
Oceania	174504	11316.1 (10935.2 to 11697)	259079	7687.3 (7466.5 to 7908.2)	-1.14 (-1.29 to -0.99)
Central Sub-Saharan Africa	1883324	14997.6 (14810.9 to 15184.3)	4152963	12629.1 (12516.5 to 12741.6)	-0.59 (-0.68 to -0.49)
Eastern Sub-Saharan Africa	3239365	7518.2 (7441.7 to 7594.6)	5666301	5489.7 (5446.6 to 5532.8)	-0.93 (-1.05 to -0.82)
Southern Sub-Saharan Africa	740067	5449.1 (5346.3 to 5551.9)	776508	3542.2 (3477.2 to 3607.2)	-1.26 (-1.39 to -1.14)
Western Sub-Saharan Africa	5321364	12195.6 (12098.5 to 12292.6)	10746979	9183.7 (9130.6 to 9236.9)	-0.81 (-0.9 to -0.73)
**Chronic hepatitis C**					
**Global**	29441837	2236.9 (2228.8 to 2245)	34003793	1738.5 (1732.7 to 1744.3)	-0.85 (-0.97 to -0.73)
**Socio-demographic index**					
High	2311014	1079.5 (1066.9 to 1092.1)	2227275	874.6 (864 to 885.1)	-0.57 (-0.65 to -0.5)
High-middle	5848012	1975.3 (1959.4 to 1991.2)	4538771	1445.3 (1432 to 1458.7)	-1.5 (-1.77 to -1.23)
Middle	10791219	2491.1 (2476.3 to 2506)	10766886	1720.7 (1710.5 to 1730.8)	-1.27 (-1.51 to -1.03)
Low-middle	7223004	2812.2 (2792.1 to 2832.4)	10097269	2016.1 (2004 to 2028.3)	-0.85 (-1 to -0.69)
Low	3245098	2889 (2859.5 to 2918.5)	6345742	2418.5 (2400.2 to 2436.8)	-0.67 (-0.77 to -0.56)

**GBD regions**					
High-income Asia Pacific	457820	967.8 (940.9 to 994.6)	247828	584.7 (562.9 to 606.6)	-2.93 (-3.93 to -1.93)
Central Asia	930500	5587.4 (5492.5 to 5682.4)	1497850	6114.6 (6029.9 to 6199.3)	0.33 (0.16 to 0.5)
East Asia	8598532	2605 (2587.5 to 2622.6)	4597039	1365.3 (1352.7 to 1377.9)	-2.67 (-3.2 to -2.13)
South Asia	4198022	1682.7 (1667 to 1698.5)	6135340	1250.1 (1240.5 to 1259.8)	-0.64 (-0.91 to -0.37)
Southeast Asia	3944738	3351 (3320.6 to 3381.3)	4827808	2617.8 (2596.3 to 2639.2)	-0.81 (-0.89 to -0.73)
Australasia	73476	1387.1 (1318.5 to 1455.6)	78359	1054.2 (1002.5 to 1106)	-0.73 (-0.88 to -0.58)
Caribbean	192028	2128.5 (2059.9 to 2197.1)	229158	1894.5 (1837.7 to 1951.2)	-0.41 (-0.44 to -0.37)
Central Europe	363654	1200.7 (1170.2 to 1231.2)	230866	857.2 (830.9 to 883.6)	-0.93 (-1 to -0.85)
Eastern Europe	1050053	1885.7 (1854.3 to 1917)	1136420	2263.2 (2226.1 to 2300.3)	0.73 (0.44 to 1.01)
Western Europe	927010	956.1 (940 to 972.3)	733047	741.4 (727.5 to 755.2)	-0.68 (-0.75 to -0.61)
Andean Latin America	105333	1179.1 (1128.6 to 1229.7)	171218	983.1 (948.5 to 1017.8)	-0.49 (-0.6 to -0.38)
Central Latin America	897666	2232.5 (2192.1 to 2273)	1198906	1750.9 (1723.3 to 1778.4)	-0.44 (-0.62 to -0.25)
Southern Latin America	81542	659.3 (627.5 to 691.2)	97765	548.3 (524 to 572.7)	-0.32 (-0.45 to -0.18)
Tropical Latin America	1010474	2630.7 (2581.9 to 2679.5)	1380998	2227.5 (2191.8 to 2263.1)	-0.07 (-0.39 to 0.25)
North Africa and Middle East	2181815	3050.3 (3011.7 to 3088.9)	3197735	2001.6 (1980.5 to 2022.6)	-1.41 (-1.5 to -1.32)
High-income North America	580521	767 (750 to 783.9)	649979	754.3 (738.5 to 770)	-0.01 (-0.12 to 0.11)
Oceania	41266	2806.9 (2636.3 to 2977.4)	97236	2809.2 (2685.5 to 2932.9)	-0.04 (-0.11 to 0.03)
Central Sub-Saharan Africa	542858	4673.2 (4573.8 to 4772.5)	1151152	3735.4 (3676.5 to 3794.2)	-0.8 (-0.9 to -0.7)
Eastern Sub-Saharan Africa	1114150	2808.8 (2764.5 to 2853.2)	2238192	2227.3 (2201 to 2253.6)	-0.8 (-0.86 to -0.74)
Southern Sub-Saharan Africa	209665	1636.3 (1583.1 to 1689.5)	365123	1684.7 (1641.3 to 1728.2)	0.05 (-0.09 to 0.2)
Western Sub-Saharan Africa	1940716	4645.3 (4586.5 to 4704.2)	3741773	3246.4 (3215.5 to 3277.2)	-1.24 (-1.39 to -1.09)

Supplementary Table 4. Prevalence of hepatitis B infection among pregnant women screened at the obstetric outpatient clinics of Jiangmen Maternal and Child Care Hospital in China, 2017 to 2023

Month	Year						
	2017	2018	2019	2020	2021	2022	2023
Jan	10.2%	8.4%	8.5%	4.1%	6.7%	7.8%	5.9%
Feb	10.3%	10.1%	7.3%	6.4%	6.3%	4.6%	5.7%
Mar	10.6%	9.5%	8.6%	7.2%	6.5%	6.6%	5.2%
Apr	8.6%	8.5%	7.4%	6.6%	7.3%	7.1%	6.2%
May	9.3%	7.8%	6.9%	7.1%	6.5%	6.1%	4.9%
Jun	10.1%	10%	6.5%	7.9%	6%	6.3%	5%
Jul	9.1%	10.1%	7.3%	8.5%	6.3%	7.6%	4.8%
Aug	7.3%	7%	10.8%	6.8%	5.3%	4.6%	2.9%
Sep	12.5%	10.5%	8.7%	8.5%	5.3%	6.6%	5.5%
Oct	8.4%	9.1%	8.8%	7.7%	6.3%	7.3%	5.5%
Nov	11.2%	8.2%	7.3%	7.7%	8.6%	4.7%	4.8%
Dec	8.9%	6.3%	8.1%	7.9%	7.3%	6.8%	4.9%
Total	9.7%	8.8%	8%	7.2%	6.5%	6.4%	5.1%

Supplementary Figure S1

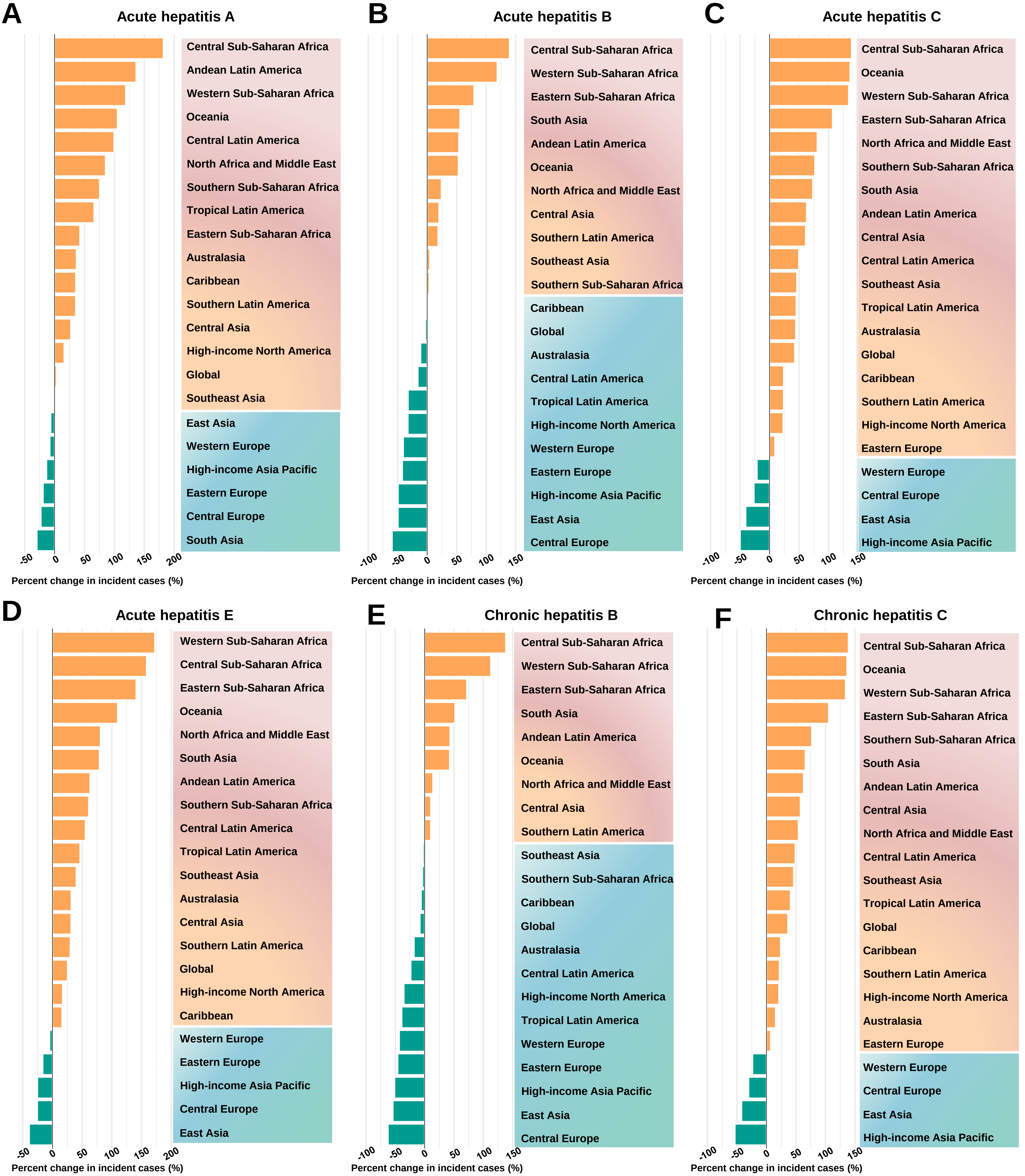

Supplementary Figure S2

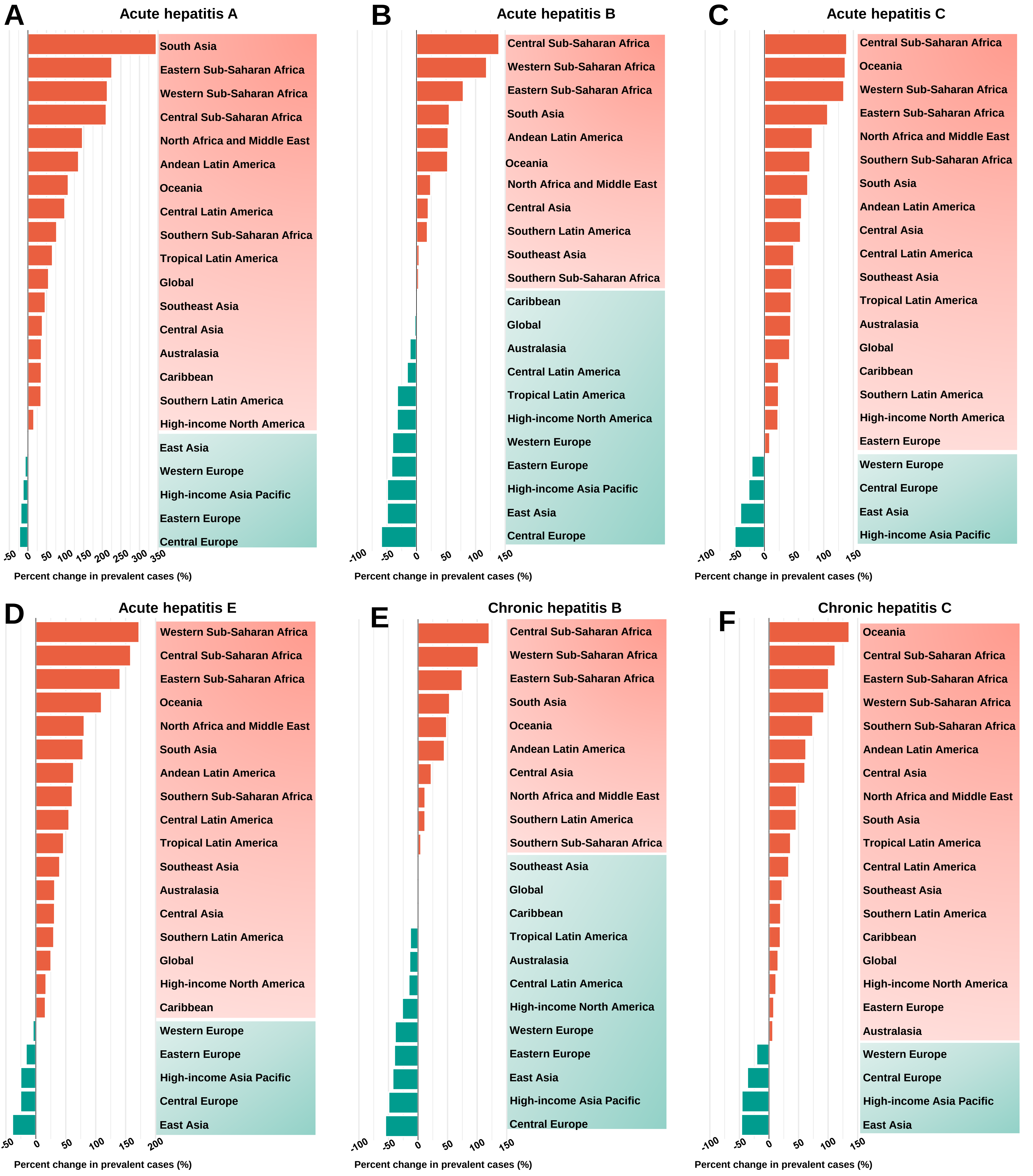

Supplementary Figure S3

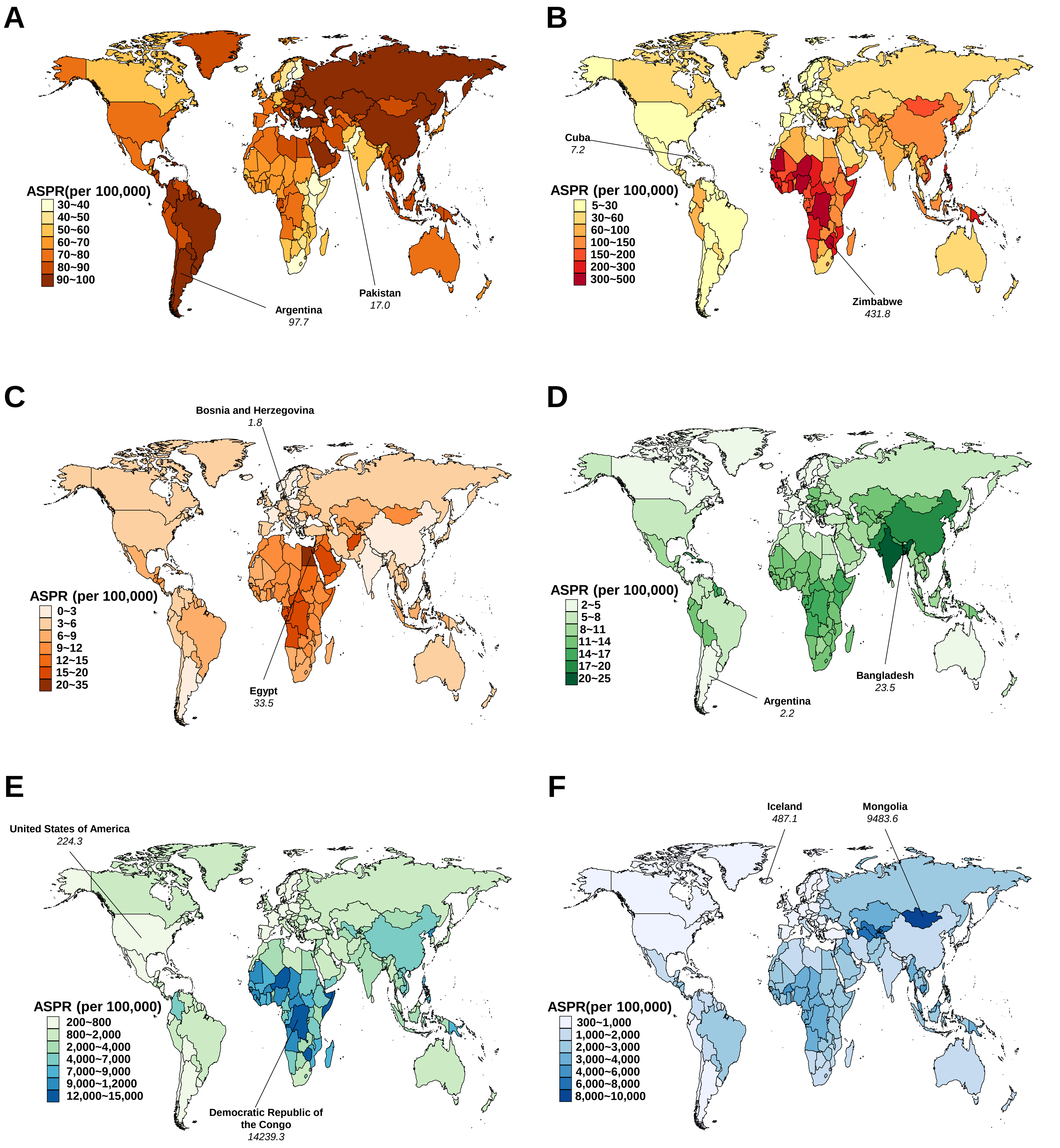

Supplementary Figure S4

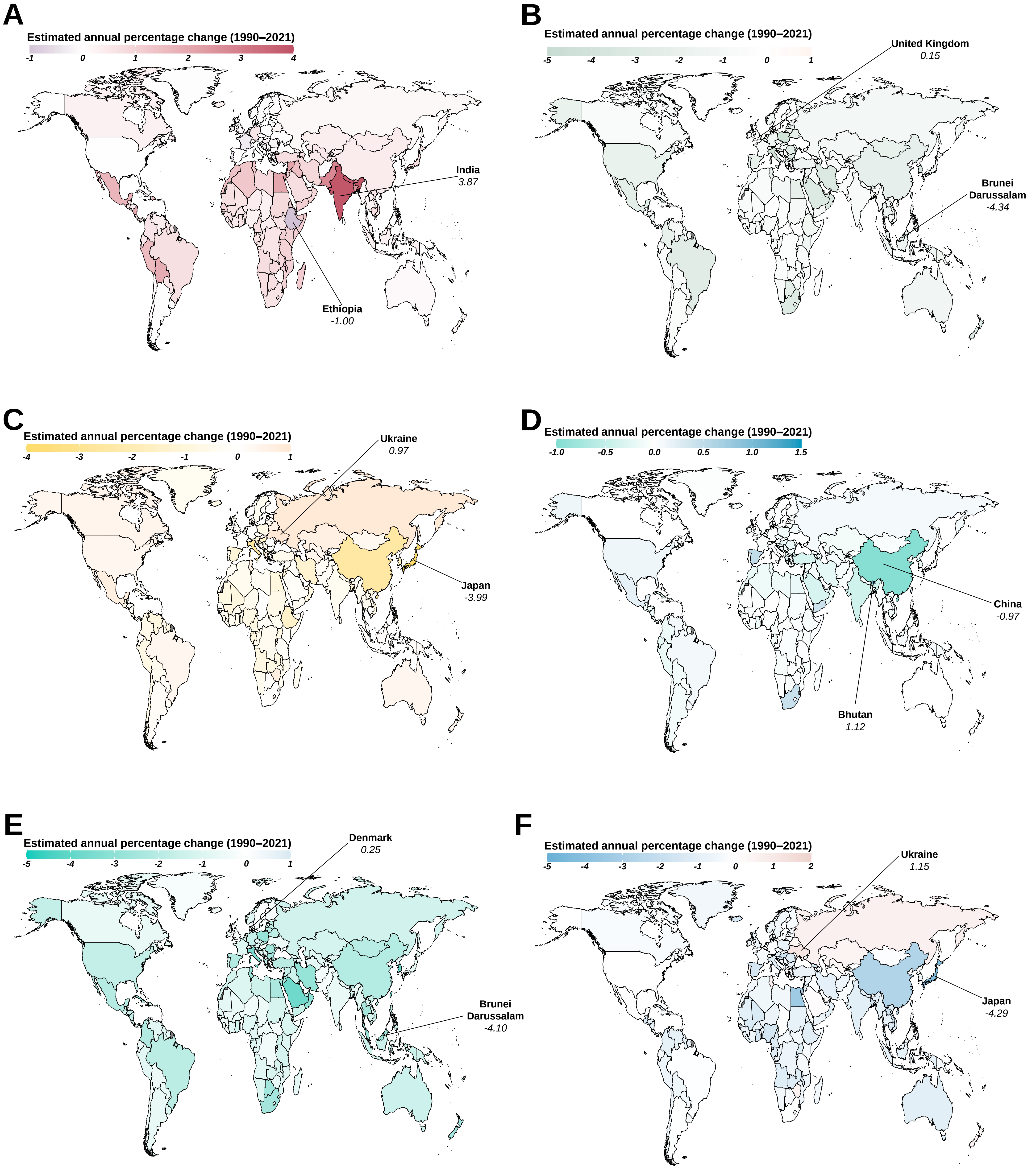

Supplementary Figure S5

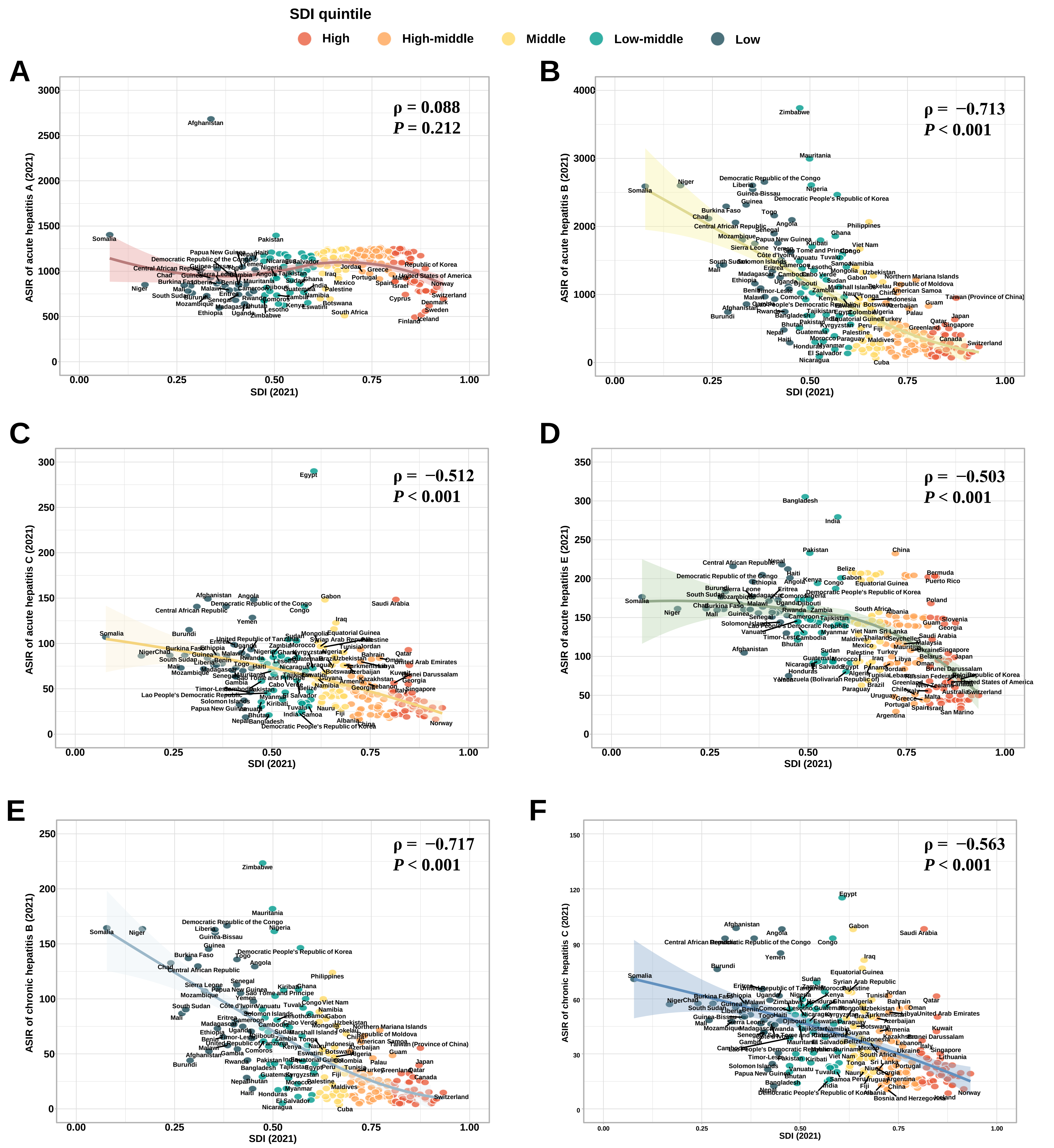

Supplementary Figure S6

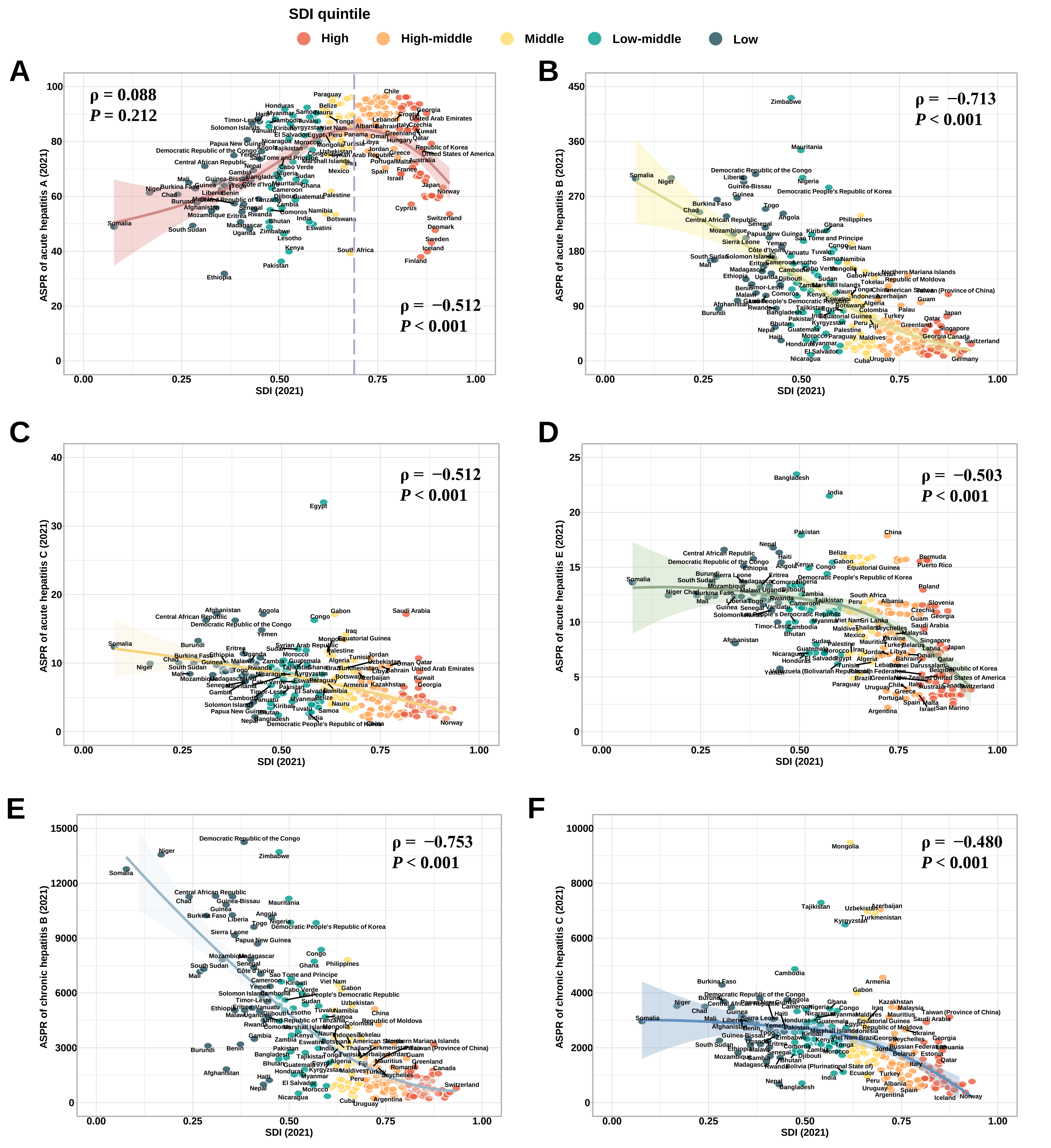

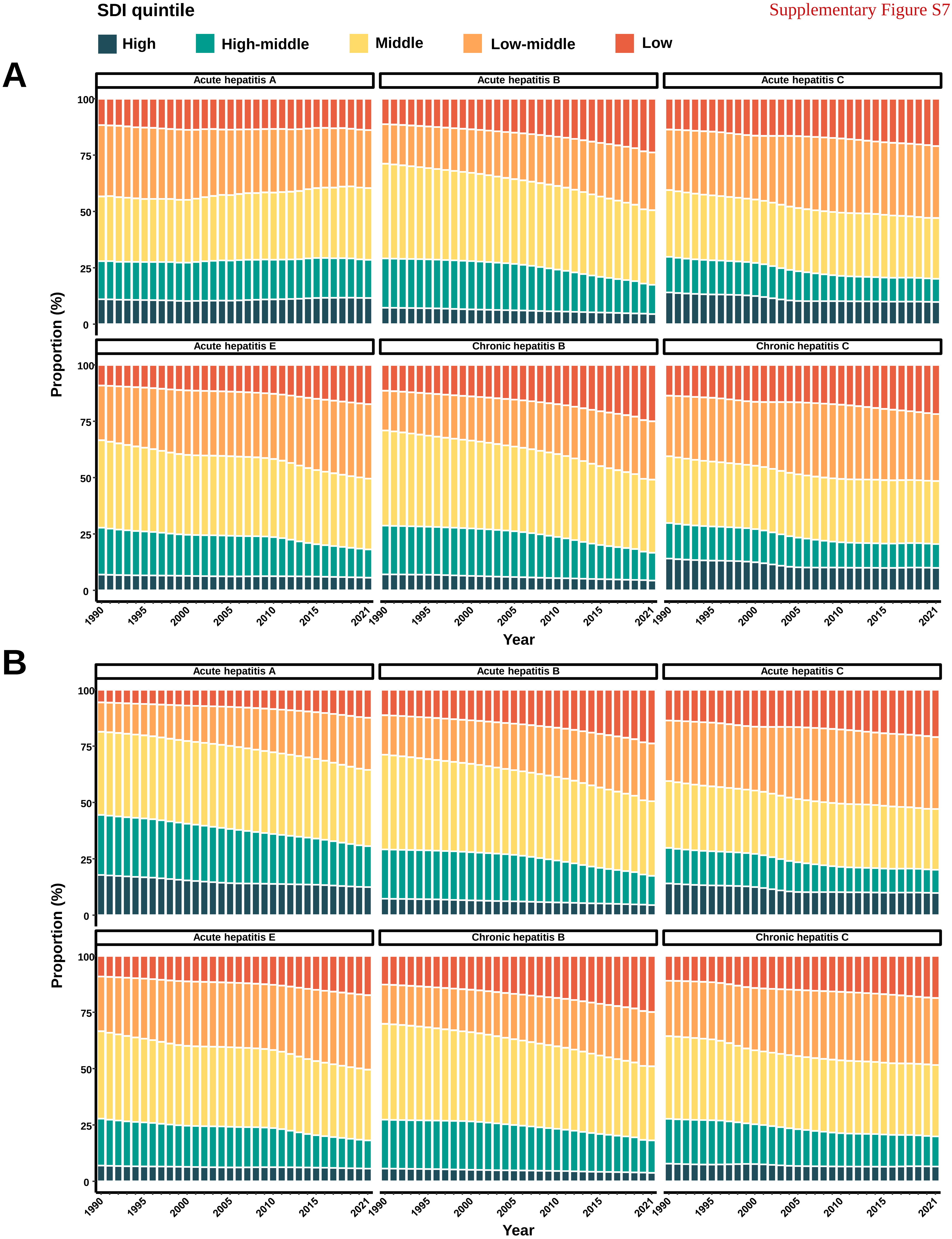
SDI quintile
High
High-middle
Middle
Low-middle
Low
A
B
Acute hepatitis A
Acute hepatitis B
Acute hepatitis C
Acute hepatitis E
Chronic hepatitis B
Chronic hepatitis C
Proportion (%)
Year
0
25
50
75
100
1990
1995
2000
2005
2010
2015
2021

Supplementary Figure S8

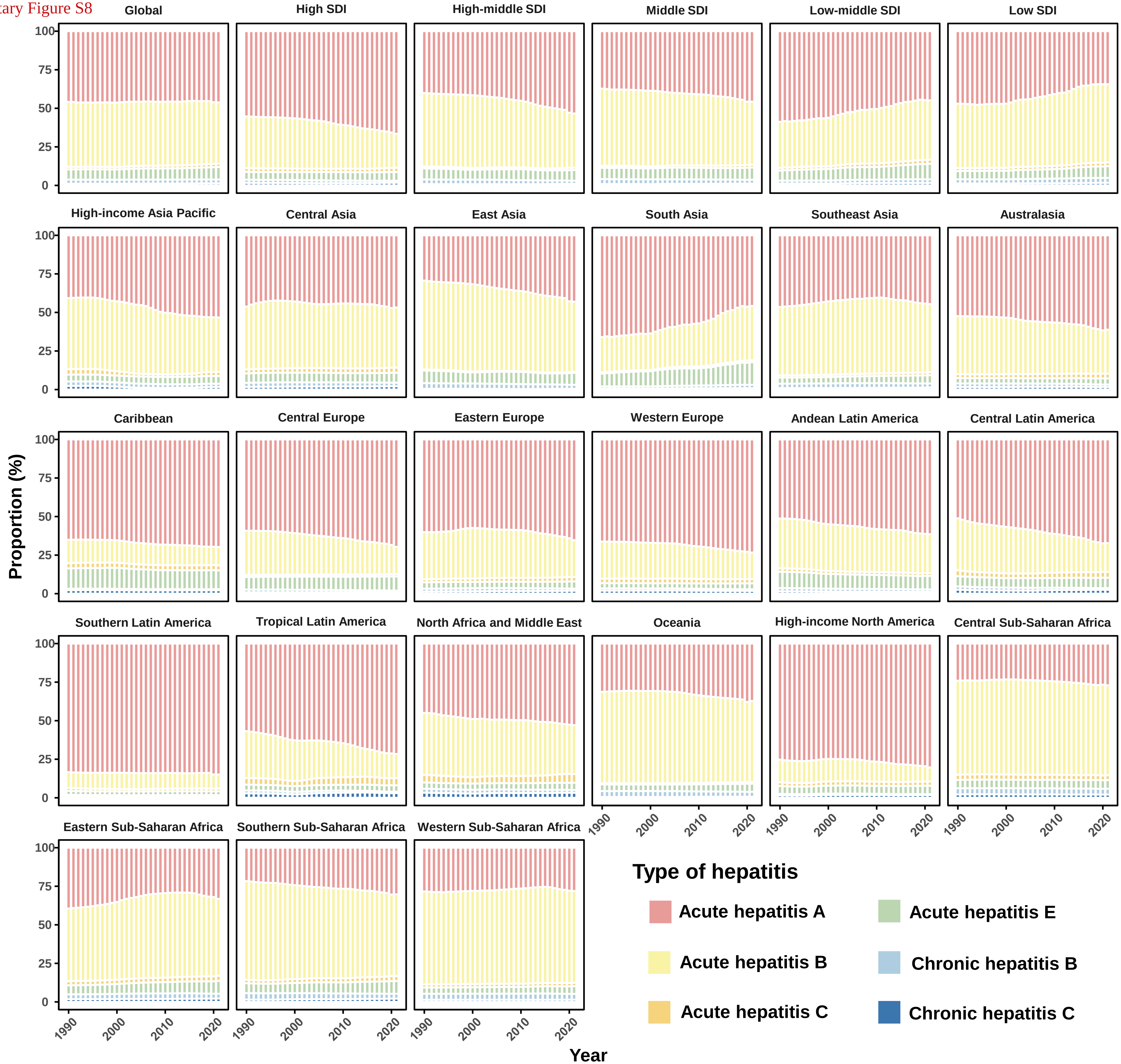

Supplementary Figure S9

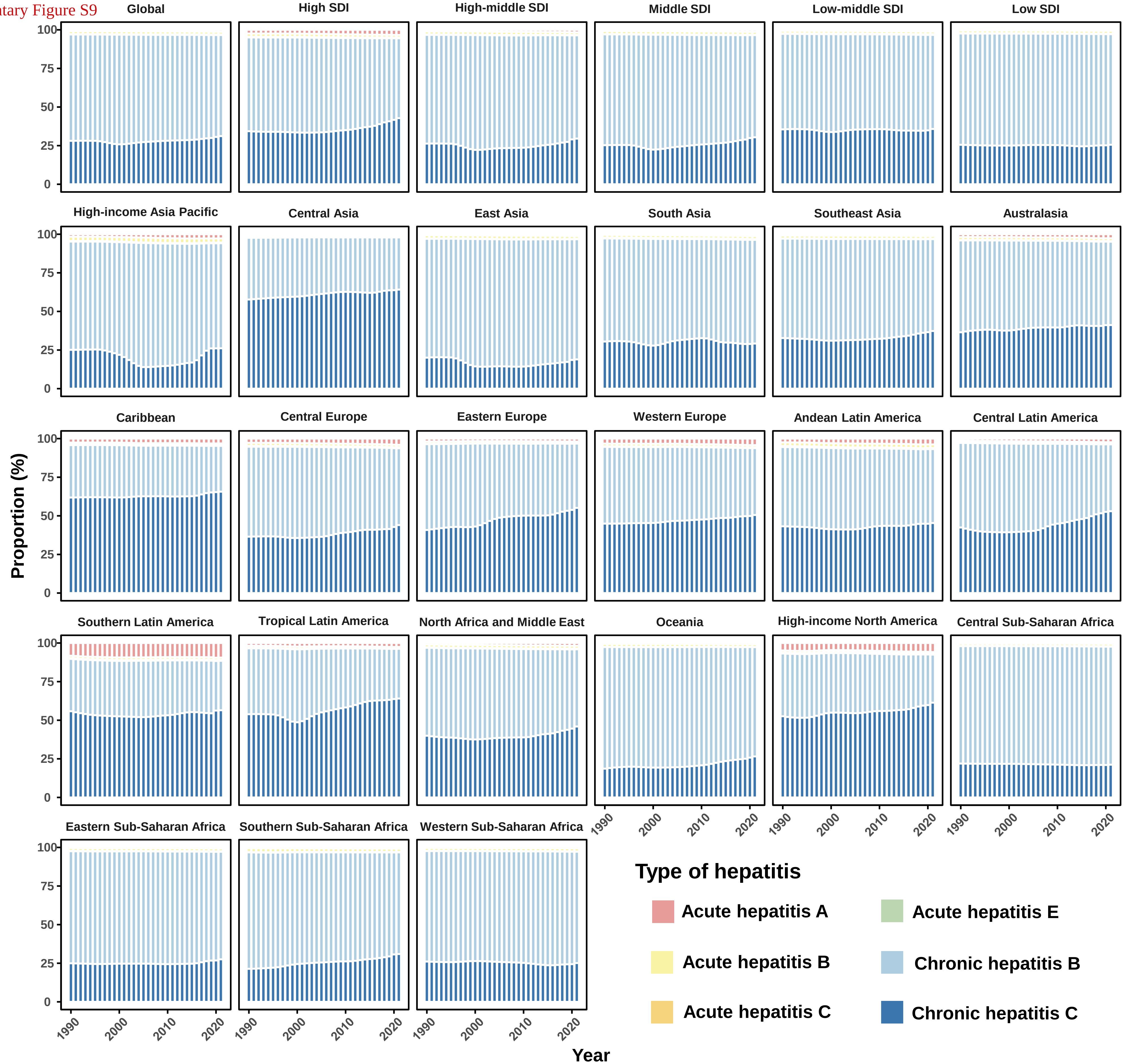

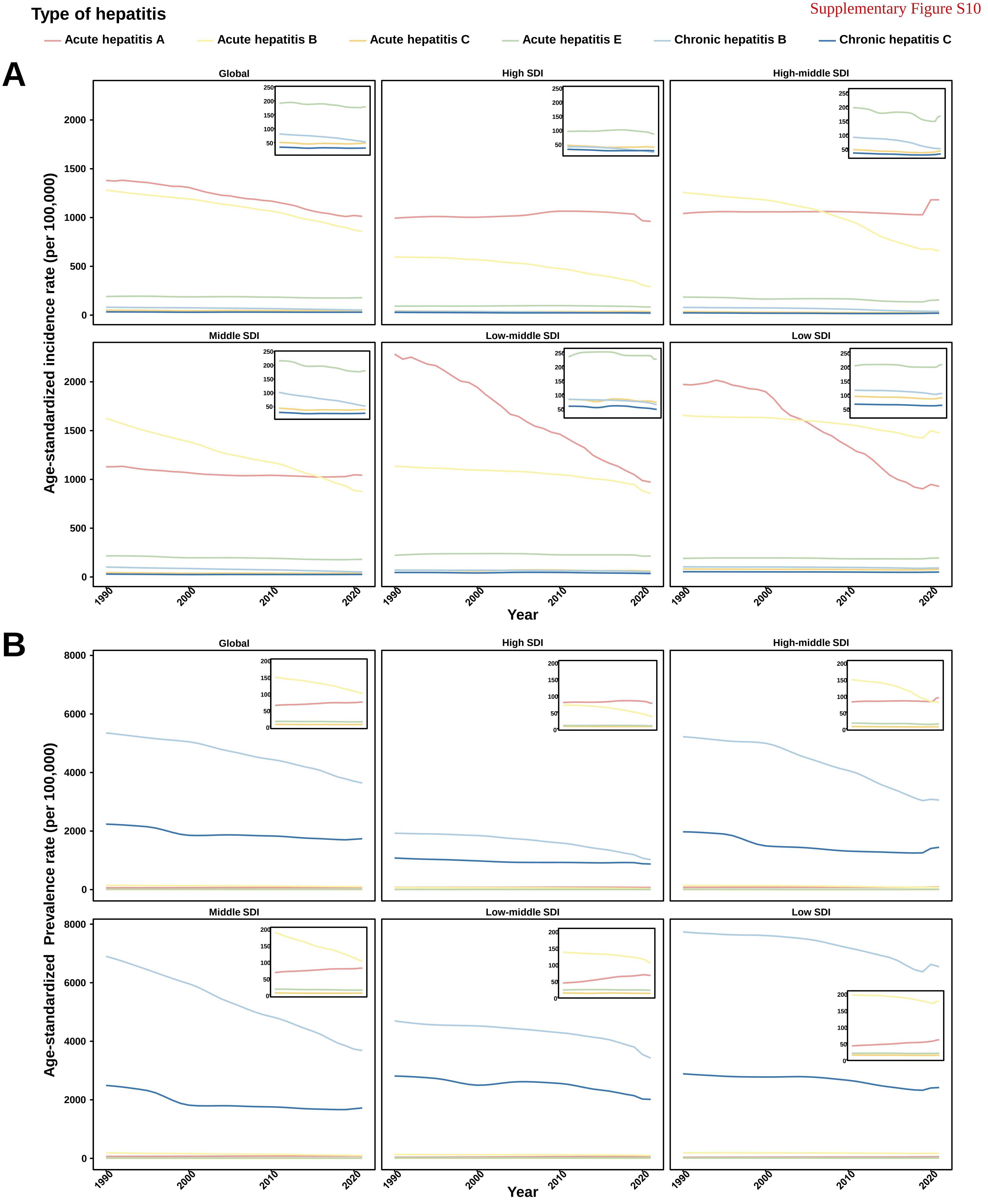
Type of hepatitis
Acute hepatitis A
Acute hepatitis B
Acute hepatitis C
Acute hepatitis E
Chronic hepatitis B
Chronic hepatitis C
A
Global
High SDI
High-middle SDI
Middle SDI
Low-middle SDI
Low SDI
Age-standardized incidence rate (per 100,000)
Year
B
Age-standardized Prevalence rate (per 100,000)
Year

Supplementary Figure S11

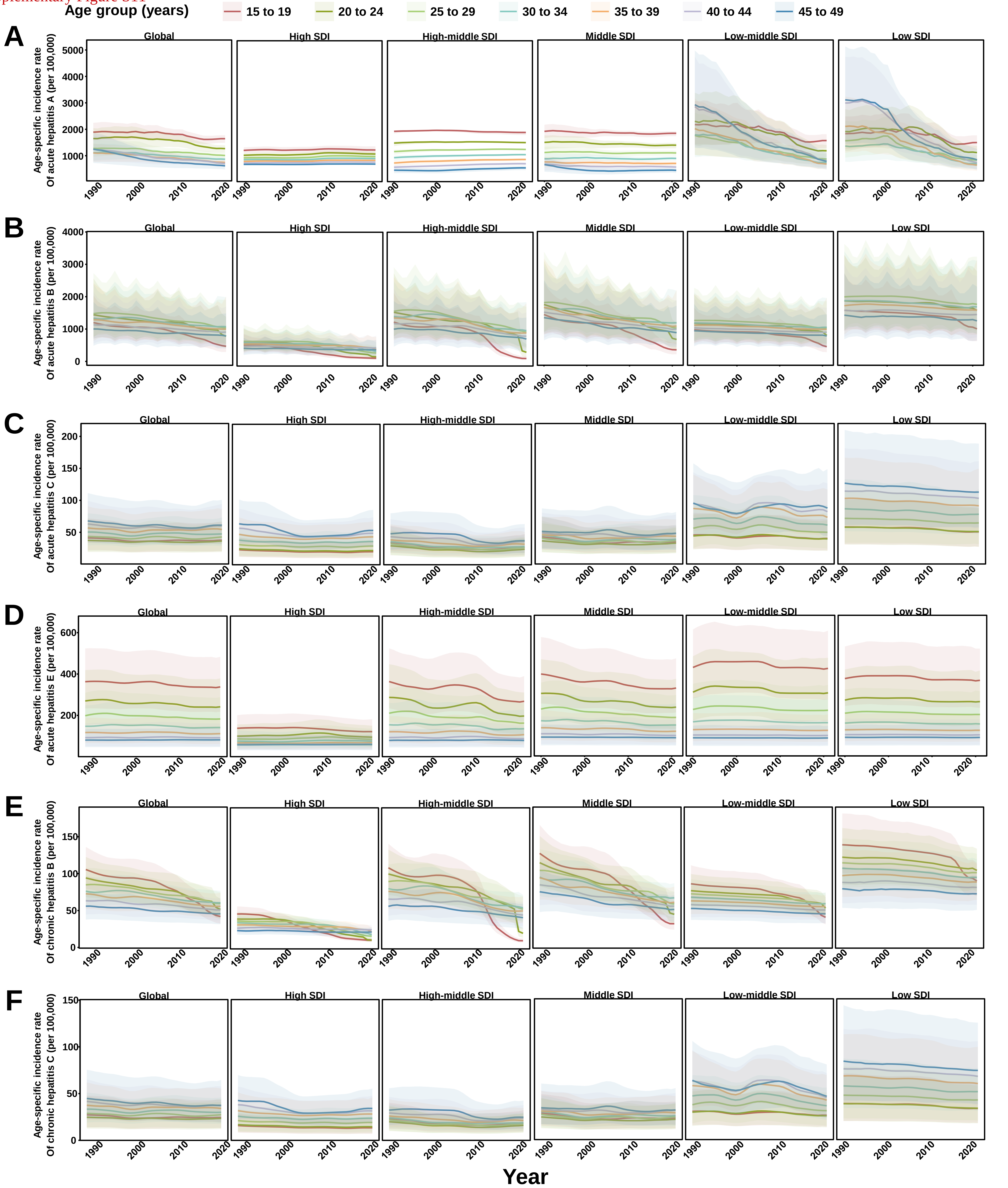

Supplementary Figure S12

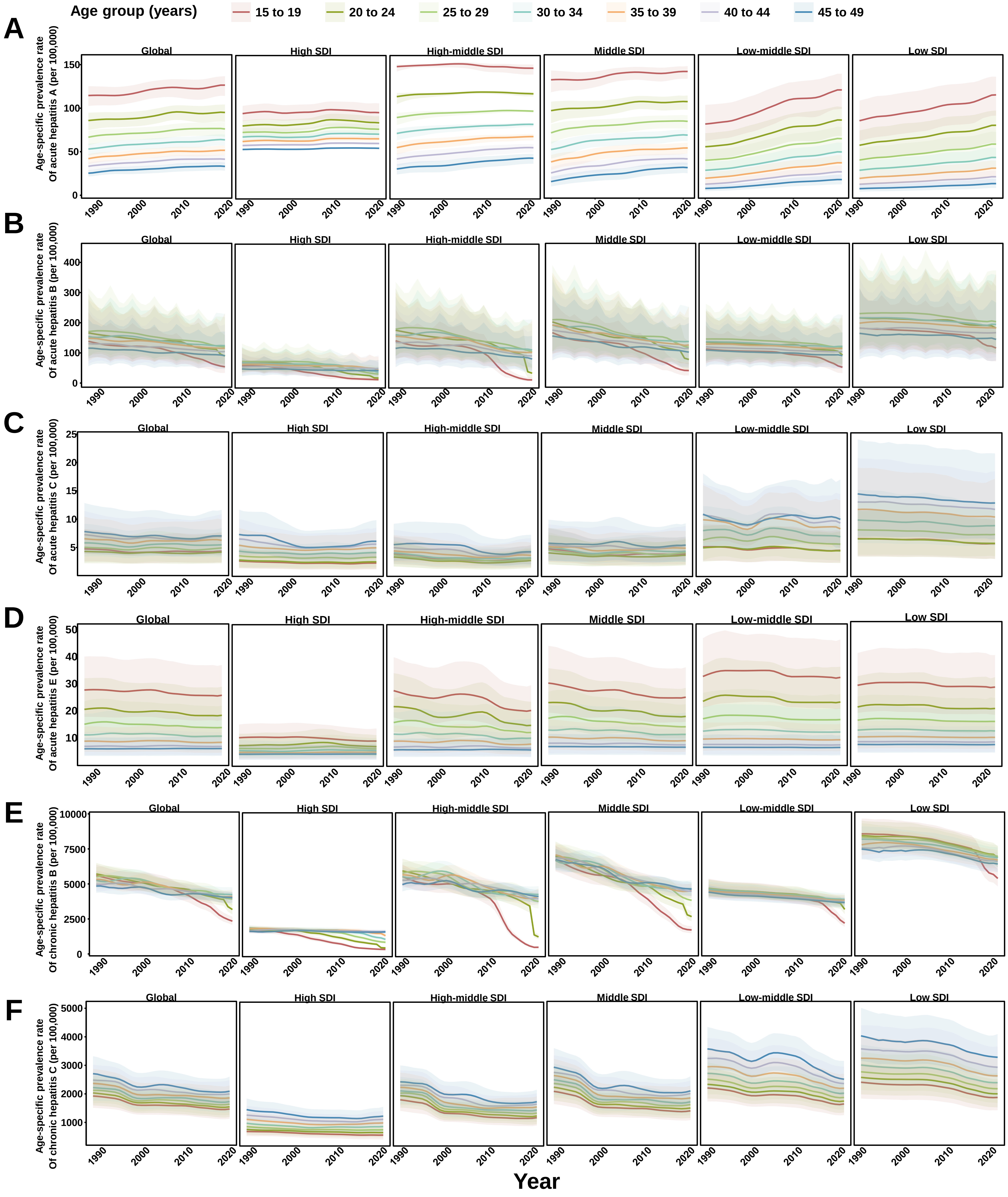

Supplementary Figure S13

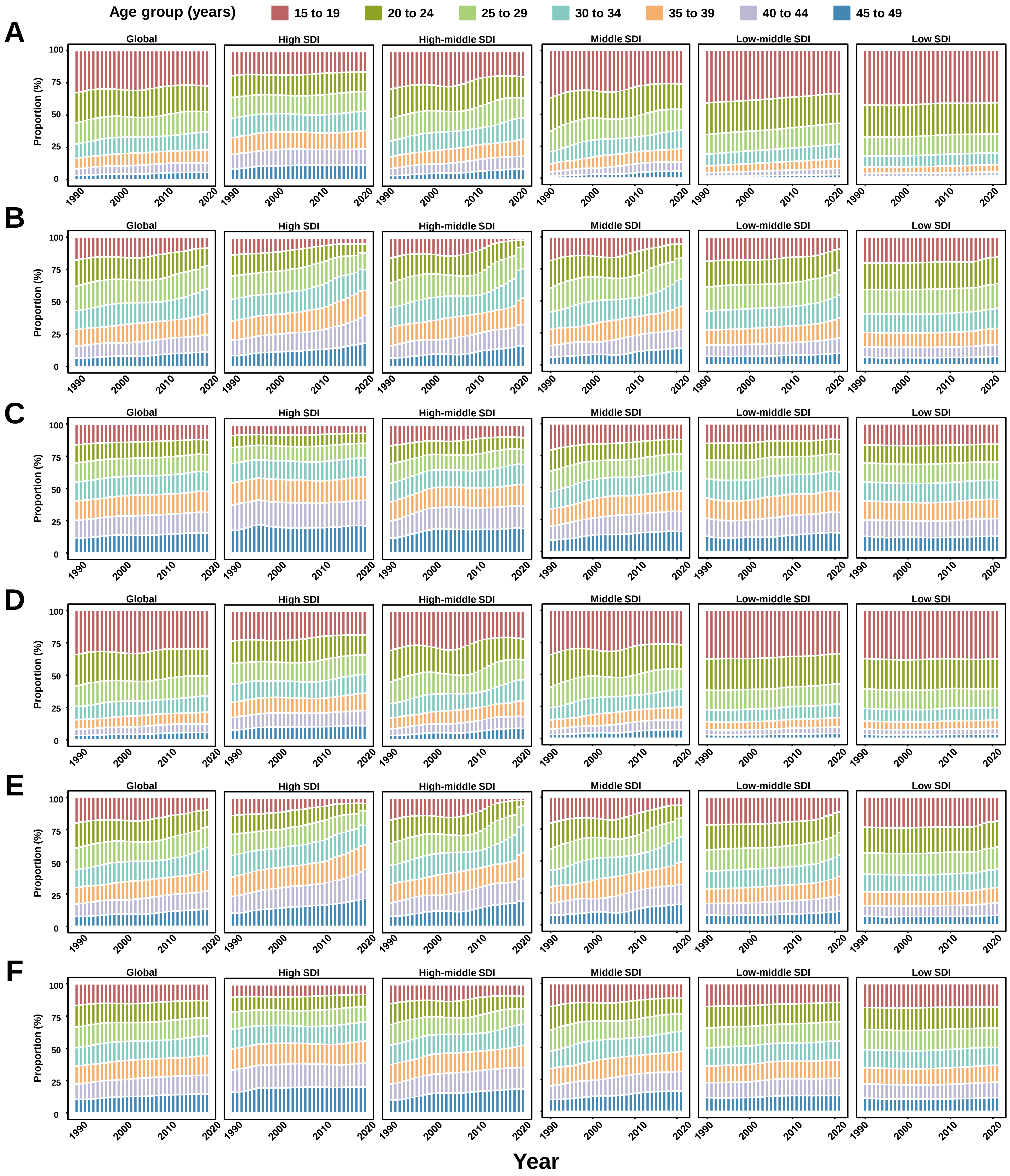

Supplementary Figure S14

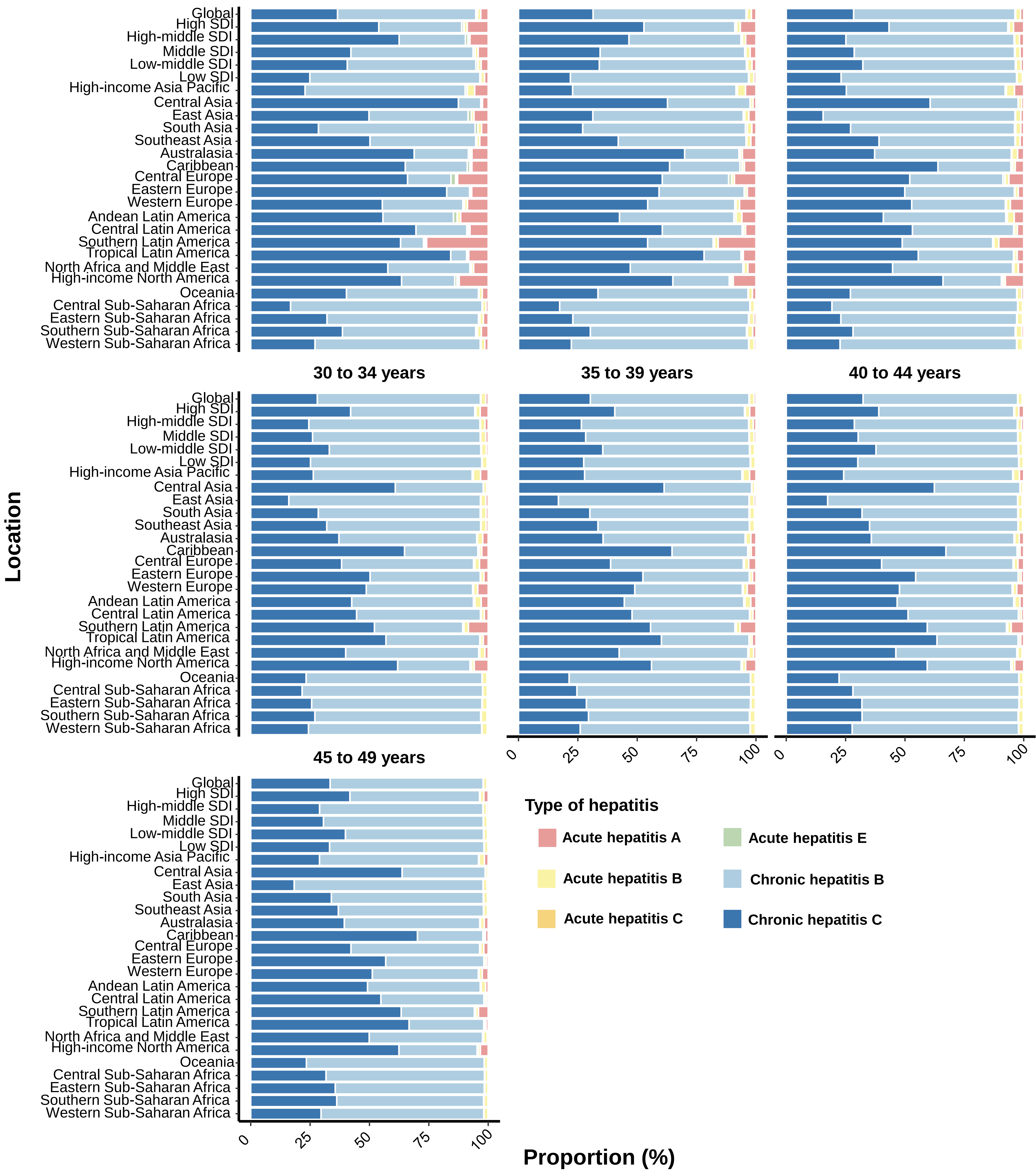

Supplementary Figure S15

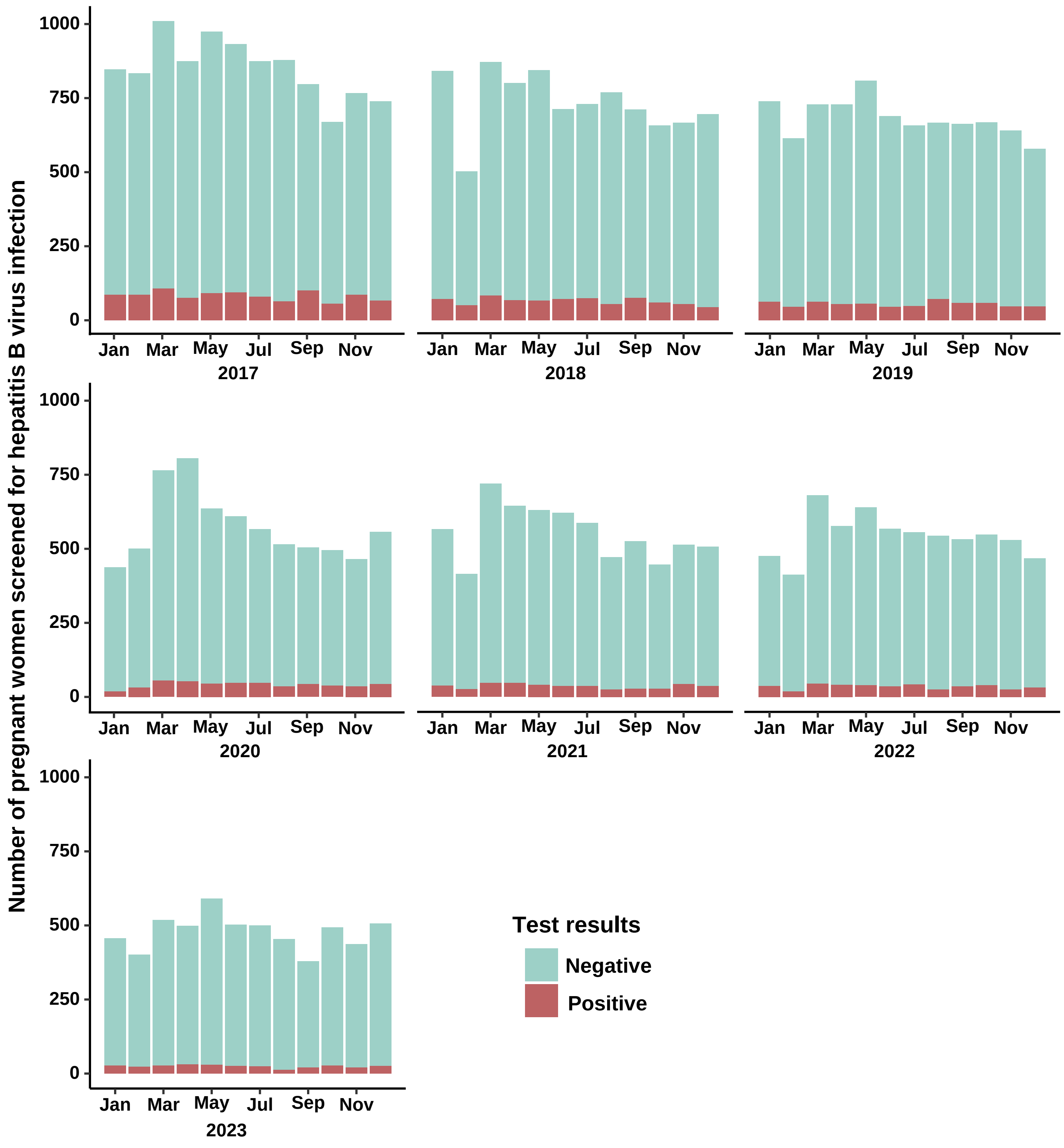

Supplement: Supplementary Figure S1 — Percent changes in incident viral hepatitis cases among women of childbearing age between 1990 and 2021, globally and across 21 GBD regions. (A) Acute hepatitis A; (B) Acute hepatitis B; (C) Acute hepatitis C; (D) Acute hepatitis E; (E) Chronic hepatitis B; (F) Chronic hepatitis C. [file Data_Sheet_1.pdf]
